# Supplementary material for: Deep cell phenotyping and spatial analysis of multiplexed imaging with TRACERx-PHLEX
Source: Nat Commun. 2024 Jun 15;15:5135. doi: 10.1038/s41467-024-48870-5 (PMC11180132; doi:10.1038/s41467-024-48870-5)
Supplement: Supplementary file 1 — Supplementary Information [file 41467_2024_48870_MOESM1_ESM.pdf]

# SUPPLEMENTARY INFORMATION

# Deep cell phenotyping and spatial analysis of multiplexed imaging with TRACERx-PHLEX

Alastair Magness*,#, Emma Colliver*, Katey S. S. Enfield*, Claudia Lee, Masako Shimato, Emer Daly, David A. Moore, Monica Sivakumar, Karishma Valand, Dina Levi, Crispin T. Hiley, Philip S. Hobson, Febe van Maldegem, James L. Reading, Sergio A. Quezada, Julian Downward, Erik Sahai, Charles Swanton#, Mihaela Angelova#

* equal contribution
# Correspondence: Alastair Magness, Charles Swanton, Mihaela Angelova

**Supplementary Methods** 3
Population and subject details 3
Imaging mass cytometry staining protocol 3
Imaging mass cytometry data acquisition 4
TRACERx Nuclear IMC segmentation dataset 4
deep-imcyto: UNet++ architecture 4
deep-imcyto: Nuclear training data augmentation 5
MCCS: Workflow overview and design 5
MCCS: Segmentation marker selection 5
MCCS: Selection of overlap, threshold and size criteria 5
TYPEx: Input cell-by-marker intensity matrix 6
TYPEx: Cell stratification by major cell lineage for cell phenotyping 6
TYPEx: Cell stratification by confidence 8
TYPEx: Cell clustering within a major cell lineage and confidence group 10
TYPEx: Detection of single-cell protein positivity with the D-score approach 10
TYPEx: Automated cell type annotation 12
Reproducibility analyses of the probabilistic models 13
Impact of noise on cell phenotyping 13
Cell phenotyping of rare cell populations 15
Barrier score validation 16
Impact of segmentation approaches on cell phenotyping and barrier score 16

**Supplementary Figures** 18
Supplementary Figure 1: Nuclear segmentation procedure implemented in deep-imcyto. 20
Supplementary Figure 2: Effects of the deep-imcyto post-processing procedure. 22
Supplementary Figure 3: The impact of the different stratification steps in TYPEx on cell phenotyping demonstrated on an example image from the TRACERx 100 IMC cohort. 24
Supplementary Figure 4: Examples of images with low and high confidence cells. 26

Supplementary Figure 5: A flowchart for automated cell assignment given a combination of expressed protein markers. 28
Supplementary Figure 6: Defining binary tissue masks to which cells can be assigned using pathologist annotations. 30
Supplementary Figure 7: Spatial-PHLEX cellular barrier quantification and validation with simulated cell position data. 32
Supplementary Figure 8: Performance evaluation of deep-imcyto compared to other segmentation models. 34
Supplementary Figure 9: Qualitative examples of deep-imcyto nuclear segmentation. 36
Supplementary Figure 10: TYPEx validation with orthogonal TRACERx data. 38
Supplementary Figure 11: TYPEx validation with public CODEX imaging data. 40
Supplementary Figure 12: Benchmarking and validation of TYPEx using public CODEX imaging datasets. 42
Supplementary Figure 13: Benchmarking of TYPEx against cell phenotyping approaches. 44
Supplementary Figure 14: Impact of different noise levels on the threshold estimate and change in cell population abundances. 46
Supplementary Figure 15: Comparison of cell phenotyping and barrier scores between different segmentation approaches. 48
Supplementary Figure 16. Pixel intensity distributions for cell subtype-specific markers across different cell subtypes, split by the confidence group. 50
**Supplementary Tables** 51
**References** 57

# Supplementary Methods

## Population and subject details

Imaging mass cytometry (IMC) data were available from 83 TRACERx patients, as well as tonsil and kidney control tissues. IMC was performed using two antibody panels: T cells & Stroma panel and Pan-Immune panel. Considering both panels, the cohort comprised of 83 TRACERx patients with 159 tumour regions from 73 patients (T cells & Stroma panel: 164 images, 144 regions, 72 patients; Pan-Immune panel: 169 images, 150 regions, 71 patients), 21 benign tumour-adjacent regions from 17 patients (T cells & Stroma panel: 24 images, 21 regions, 17 patients; Pan-Immune panel: 19 images, 17 regions, 13 patients), five lymph node regions with no tumour content (T cells & Stroma panel: 5 regions, 5 patients; Pan-Immune panel, 4 regions, 4 patients), one lymph node region with tumour content (T cells & Stroma panel and Pan-Immune panel), 49 adjacent normal lung cores from 49 patients (T cells & Stroma panel: 50 images, 46 regions, 46 patients; Pan-Immune panel: 51 images, 46 regions, 46 patients), tonsil controls (T cells & Stroma panel: 29 images; Pan-Immune panel: 33 images), and one kidney control (T cells & Stroma panel and Pan-Immune panel). Further, we included data from a single tumour region that we were unable to map to patient metadata. Multiple images per region resulted from technical replicates or where multiple Tissue MicroArray (TMA) cores were taken from the same regional FFPE block and inserted into independent TMAs.

## Imaging mass cytometry staining protocol

Tissue sections of TRACERx TMAs and control tonsil tissues were cut from FFPE blocks at a thickness of 5µm and floated onto standard positively charged slides for immunostaining. Sections were baked at 60°C in preparation for antibody staining. Sections were dewaxed in xylene (2x 5 min) and rehydrated in a graded series of alcohol (ethanol:deionized water 100:0, 100:0, 70:30, 0:100, 0:100, 1 minute each). Heat-mediated antigen retrieval was conducted in Tris-EDTA buffer at pH 9 for 30 minutes in a 900W microwave. Tissue sections were slowly cooled in a room temperature water bath to prevent buffer crystallisation. Sections were then washed in 1X PBS, and the tissue area was circled with a hydrophobic PAP pen (Vector Laboratories Inc, Oxfordshire, UK, catalog number H-4000) to create a reagent barrier. Samples were dipped briefly in PBS-Tween20 (0.2%) (Sigma-Aldrich (Merck), Dorset, UK, catalog number P1379), before blocking with 1% BSA/PBS for 30 min at room temperature. Excess blocking solution was flicked off and the solution of primary antibodies (diluted in 1% BSA/PBS) was applied and left overnight at 4°C (**Supplementary Tables 1-2**). The next day, sections were washed three times in PBS (1 min each), dipped briefly in PBS-Tween20 and

counterstained with iridium (1:500 in PBS, Fluidigm, San Francisco, USA) for 30 minutes at room temperature. Samples were washed three times in PBS (1 min each) and counterstained with ruthenium (1:1000) for 5 minutes on ice in a fumehood[1]. Samples were washed three times in milliQ water (1 min each) and left to air dry.

**Imaging mass cytometry data acquisition**

IMC data from TRACERx and control FFPE samples were acquired using a Hyperion Imaging System using commercial Fluidigm IMC software (version 6.7). Regions of interest (ROIs) were designed to capture each full TRACERx TMA core (1.5mm diameter) or 1.0mm diameter ROIs of control tonsil tissue. A laser ablated tissue ROIs in a rasterized pattern at 1µm resolution and 200Hz. The instrument was tuned between each run using a 3-Element Full Coverage Tuning Slide (Fluidigm, San Francisco, USA, PN 201088).

We recommend establishing a system to annotate .mcd file names and the selected regions of interest (ROIs). In the TRACERx study, we made sure to avoid spaces and special characters in the .mcd file names, which may result in errors when running PHLEX. We also established a mapping file for our TMAs, such that the ROIs could be labelled with a simple name within data acquisition software (e.g. TMA001_A_1 for TMA, row, column) that could be linked back to more detailed sample labels and metadata for data analysis.

**TRACERx Nuclear IMC segmentation dataset**

The TRACERx nuclear IMC segmentation dataset (TRACERx NISD) comprises nuclear labels from 116 256x256 µm image tiles, including lung adenocarcinoma (LUAD) (n=29 cores, 40 tiles), lung squamous cell carcinoma (LUSC) (n=21 cores, 33 tiles), other non-small cell lung cancer (NSCLC) (n=7 cores, 9 tiles) histological subtypes, histologically normal lung tissue (n=14 cores, 17 tiles), tumour cores reclassified as benign tumour-adjacent following pathology review due to a lack of tumour content (n=9 cores, 9 tiles), one lymph node resected at primary surgery with no tumour content (n=1 core, 1 tile), tonsil (n=5, 5 tiles), and one kidney sample (n=1 core, 2 tiles). It is, to our knowledge, the largest IMC-specific nuclear segmentation dataset currently available. The TRACERx NISD can be used in isolation, or in conjunction with other IMC datasets to train state-of-the-art nuclear segmentation models for IMC images.

**deep-imcyto: UNet++ architecture**

UNet++ is a modification of the common U-net segmentation architecture with additional skip connections between convolutional layers, and the $L^4$ variant is based on a core U-net of four

downsampling and four upsampling convolutional layers. It has shown improved performance over U-net in a variety of medical image segmentation tasks[2].

### deep-imcyto: Nuclear training data augmentation

Data augmentation during deep-imcyto UNet++ model training included performing flips, scaling, translation, shear, and rotation transformations of images and masks. With IMC being a particularly noisy imaging modality, we placed added emphasis on noisy augmentations of ground truth, applying some degree of additive Poisson, Gaussian or Laplacian noise to 75% of training images.

### MCCS: Workflow overview and design

MCCS first segments whole cells using a user-curated set of cell lineage markers ('segmentation markers'), one marker at a time independently of one another to create whole cell masks for each segmentation marker. The whole cell segmentation mask is then created from the consensus of these individual masks. In this way, MCCS aims to more fully harness the highly multiplexed nature of IMC data by not collapsing different marker channels into a single channel prior to cell segmentation.

As in our TRACERx study, an additional primary object identification step can be added to identify non-nucleated cell content for a marker of the user's choice and resulting cells combined with those from the consensus nucleated mask to generate a final whole cell mask.

Should a new user wish to apply an MCCS workflow to their own dataset, a template MCCS procedure is distributed as part of PHLEX, and we provide detailed guidance on creating an MCCS pipeline in CellProfiler in the TRACERx-PHLEX online documentation.

### MCCS: Segmentation marker selection

The set of segmentation markers chosen (**Supplementary Table 4**) comprised those with high signal-to-noise ratio and specificity, low prevalence of artefacts, and representing the greatest number of distinct cell lineages for the respective antibody panel, and were assessed over a representative subset of TRACERx lung images.

### MCCS: Selection of overlap, threshold and size criteria

For the creation of segmentation marker cell masks, an overlap criterion that at least 50% of nuclear edge pixels should overlap with the segmentation marker mask was selected and validated by visual inspection. This overlap criterion was implemented to address the observations that (i) we frequently saw overlap of lineage markers with pixels at the edge of

segmented nuclei, (ii) we would not expect cytoplasmic/membrane markers to overlap with central nuclear pixels, (iii) the observation that, even in cells characterised by a segmentation marker, there is not 100% overlap with nuclear edge pixels.

During identification of non-nucleated αSMA$^+$ fibroblast content, we filtered the identified non-nucleated cells to remove those with a large degree of overlap with nucleated cell objects (non-nucleated cells with <⅓ area overlapping nucleated cells retained) and by size (cells of >20 pixels following masking by the nucleated cell mask retained). These steps were implemented to reduce the likelihood of double counting cells and of assignment of cell debris or artefacts to cell area.

Selection of the aforementioned thresholding, overlap and size parameters were optimised qualitatively by visual inspection across a range of cores, adjusting CellProfiler parameters accordingly. The authors note that such validation was time-consuming.

**TYPEx: Input cell-by-marker intensity matrix**

The input cell-by-marker matrix can be populated with any value that summarises the intensity of a marker per cell object, and it is independent of the imaging modality or antibody tagging technique. The intensities of the deposited Barrett's Esophagus (BE) and HuBMAP datasets were originally normalised and included negative values. For TYPEx analysis, the absolute minimum intensity value was added to all intensity values in a dataset to ensure only non-negative values in the cell-by-marker table.

**TYPEx: Cell stratification by major cell lineage for cell phenotyping**

TYPEx involves four analytical components: cell stratification, detection of protein expression, automated cell annotation and tissue segmentation. The cell stratification involves three steps: stratification by major cell lineage, stratification by confidence and clustering.

The cell stratification steps aim to find groups of cells characterised by a distinct protein expression profile and assign them to low- and high-confidence groups. To stratify by major cell lineage, TYPEx creates a probabilistic model that annotates each individual cell using CellAssign[3], based on the major cell lineage definitions provided as input config file (cell type annotations). CellAssign is specified with the argument *--major_method* and is currently the only probabilistic method implemented to perform this step. CellAssign leverages the hierarchy of cell lineages as additional information to the model.

CellAssign was originally developed for single-cell RNA sequencing data and uses raw read count as input. It assumes that the cell-type specific markers have significantly higher

intensities in the cell types they identify compared to cell types they do not identify, and the levels of these marker intensities can vary between different markers. To configure CellAssign for analysis of pixel intensities, TYPEx skips the normalisation by library size and sets the cell-specific size factor to one.

In addition, the raw marker intensities were multiplied by a factor of ten. The factor 10 was chosen from several evaluated factors (1, 10, 100), by comparing the proportion of CD4 and CD8 major cell lineages in the probabilistic model to the expected T cell proportions $CD4^+$ and $CD8^+$ in the TRACERx cohort derived from orthogonal flow cytometry datasets. The default value is applicable to raw input cell-by-marker intensity tables and deep-imcyto in *simple* mode. The default factor of ten was also applied to the z-score normalised intensities in the HuBMAP and BE datasets.

If the input intensities are rescaled in the range 0-1, the argument *magnitude* in the config file *typing params* should be used to set the scaling factor. The intensities in the cell-by-marker matrix generated by deep-imcyto using our MCCS procedure in *CellProfiler* mode needed to be further multiplied by a factor of $10^5$ to be scaled back to the raw pixel intensities. The intensities output by MCCS are scaled in the range from 0-1 due to its CellProfiler implementation. MCCS rescales the intensities of input images for all channels, dividing by a user-defined number exceeding the maximum intensity in the dataset, $10^5$ , to ensure that the intensity values fall in the range 0-1 for the two IMC TRACERx datasets. Notably, the intensity distributions remain untransformed. This refactoring is implemented to meet a requirement from CellProfiler that all saved .tiff files output from the *full_stack_preprocessing* step should have intensity ranges between 0-1. Therefore, for all analysed datasets, the parameter *magnitude* was set to the recommended default value of 10, except for the TRACERx data analysed with the MCCS procedure, where *magnitude* was set to $10^6$ (*i.e.* magnitude $10 * 10^5$).

CellAssign also accounts for batch effects, which can be considered if provided in a sample-annotation table and specified as input parameters to TYPEx for batch correction in the *typing params* config file.

We allowed CellAssign to allocate the most likely cell type for each cell, even in cases of low probability scores (configured to any *assign_prob*). Even when the model could not determine a cell type with high probability, it assigns the most likely cell type, for example Epithelial cells in the case of the T cells & Stroma antibody panel. TYPEx then leverages the probability information to minimise ambiguous cell type calls by further stratifying the cells by confidence. Building the probabilistic model can be a time-consuming process depending on the sample size and number of major cell-lineage markers; therefore, high performance computing is recommended for this step.

**TYPEx: Cell stratification by confidence**

Signal spillover into the neighbouring cells and low signal-to-noise intensities were the key challenges that motivated the development of TYPEx. Lower confidence cell assignments are often of tightly packed cells, for example those in lymphoid aggregates, or have weak marker intensities and low signal-to-noise ratio (**Supplementary Fig. 3, Supplementary Fig. 4**). We have illustrated examples of the median raw pixel intensity across low- and high-confidence cells of a given cell type per image in **Supplementary Fig. 16**.

In lung tissue and tonsils, we frequently observed areas of densely packed immune cells. Using the clustering approaches implemented in Phenograph and FastPG, we identified a high proportion of double-positive $CD4^{+}CD8^{+}$ T cells in the TRACERx 100 IMC dataset with the T cells & Stroma panel (42% and 66%, respectively, **Fig. 7g**). Generally, across all cell subtypes in the T cells & Stroma panel, we observed 17%-28% ambiguous assignments (**Fig. 7f**).

One of the steps that TYPEx undertakes to resolve the high ambiguity in cell phenotypes observed with clustering approaches is stratification of the cells into low and high confidence. TYPEx relies on the assumption that the spilled over signal is weaker in the neighbouring cell than the cell from which the signal originates. Therefore, when the signal of the defining markers is strong in the original cells, these cells will be assigned as high confidence cells despite the spilled over signal. For example, **Supplementary Fig. 3** zooms in on one example region with many closely packed T cells, B cells and macrophages, which have been identified as low confidence cell assignments. Using the clustering approach FastPG, most of these cells have been classified as Ambiguous. In this example, 43% of the T cells were identified as double-positive $CD4^{+}CD8^{+}$ T cells. Using TYPEx, the proportion of $CD4^{+}CD8^{+}$ T cells was reduced to 7.5% in this image. Overall, **Figure 7g** demonstrates that the stratification steps significantly reduced the number of detected double positive $CD4^{+}CD8^{+}$ T cells in the cohort.

To differentiate between low and high confidence assignments, TYPEx seeks to identify the cell annotations that remain stable and those that vary when providing partial information to the probabilistic model. The rationale behind this approach relies on the assumption that robust cell annotations are more likely to retain their label when perturbing the input information, whereas the low confidence annotations are more likely to change their label. Therefore, by excluding a major cell lineage and the markers associated with it, the model will be given less information, which results in all cells from the excluded cell lineage to change labels. In addition, we expect that a subset of cells assigned to other major cell lineages with

lower confidence also change their label. Labels concordant between the complete and incomplete model are considered stable annotations.

The stable and variable annotations would ideally be defined by perturbing the model with exclusion of different cell lineages. However, building the probabilistic model is a time-intensive process (**Supplementary Table 3**). Therefore, building multiple models to exclude different cell lineages is computationally expensive. To circumvent this issue, we run the incomplete model only once but use the properties of the varying labels to predict low confidence cells based on their probability scores and the mean intensity of lineage-specific markers. TYPEx builds a model to distinguish between stable and varying annotations by their probability score and mean intensity. This binomial logistic regression model is used to predict and classify low and high confidence cells. The fitted model is then applied to classify the cells in the cohort into low and high confidence based on their probability scores and lineage-specific marker intensities. The model is applied on all objects (including the undetermined).

A binomial logistic regression was built given the major cell-lineage assignment probability and mean intensity of the corresponding cell type-specific markers, as

$$stability\_status \sim probability + meanIntensity + (1|cellType) \qquad (1)$$

where the *stability_status* is determined by runnung an incomplete probabilistic model with CellAssign with one major cell lineage is excluded as the status of the assignments consistent with the complete model - likely true and the discordant - likely false. For example, Mesenchymal cells were excluded from the T cells & Stroma panel (Vimentin) and Myeloid cells from the Pan-Immune panel (CD11b).

To automate this process, TYPEx was designed to exclude the most frequent cell type from the complete model. To do this, it waits for the complete model to be built in order to start with the process that builds the incomplete model with the excluded cell lineage. To parallelise these processes, the parameter *–exclude_cell_lineage* can be used to specify this cell lineage and both models will be built in parallel when the resources allow.

When the probabilistic model built with CellAssign cannot confidently assign a cell type to a given cell, it assigns the most likely cell type to this cell. Therefore, when removing the most likely cell type and the marker specific for that cell type from the model, we build the incomplete model where all the low confidence cell types will be assigned to the next most frequent cell type in the user-provided definitions. Therefore, all the low confidence cell type calls will change the label between the complete and incomplete model.

Specifically for the example with Vimentin, when removing the Vim+ cells and Vimentin from the dataset, there won't be any marker in the dataset that can confidently identify these mesenchymal cells. They will be assigned to the most likely cell type as well and consequently change their label.

Finally, to evaluate how robust the probabilistic models are, we compared the major cell lineage assignments with CellAassign between three iterations of random subsampling (~2.1/3.16 million cells, **Supplementary Fig. 13a**). The CellAssign annotations reached a concordance of 91-95% among the subsampling runs.

In general, if there are a lot of cells changing labels, this number would reflect the proportion of the most frequent cell type in the dataset. However, if there are a lot of cells from the remaining cell types that are identified as low confidence, this may indicate that the cell type definitions do not cover the major cell lineages in the dataset. For example, there is no epithelial marker to cover the epithelial population in the dataset.

**TYPEx: Cell clustering within a major cell lineage and confidence group**

TYPEx splits the cells by major cell lineage and confidence group to perform clustering. For each group of cells, for example, low-confidence Epithelial cells, it runs the selected clustering approach. The TYPEx workflow allows for the user to specify any of the clustering approaches for cell stratification. The following clustering approaches are currently implemented to be selected: FlowSOM[4], FastPG[5] and Phenograph[6]. TYPEx allows flexibility, in that the stratification steps can be skipped and only clustering run followed by protein expression detection and annotation. TYPEx allows the possibility to perform the tiered TYPEx approach (*--tiered* true) with or without stratification by confidence (*--stratify_by_confidence*), including clustering alone (*--clustered*) or sampling of the data (*--sampled*).

**TYPEx: Detection of single-cell protein positivity with the D-score approach**

To avoid using a global cutoff on intensities, which can be affected by technical and biological factors, we transform the marker intensities of a given cluster to a D-score in the range 0 to 1 for each marker. A D-score is calculated for each cluster within the corresponding confidence population. For example, the D-score for a given cluster of low-confidence Epithelial cells is calculated by pairwise comparison of this cluster to all clusters from the low-confidence population (regardless of the major cell lineage), whereas the D-score for a cluster from the high-confidence Epithelial cells is calculated by pairwise comparison to all clusters from high-confidence populations (regardless of the major cell lineage).

To perform automated marker detection, TYPEx relies on a subset of markers which are co-expressed and mutually exclusive with markers in the antibody panel. These markers are only used to identify the optimal D-score cutoff. The D-score cutoff is determined so that the rare cell populations' frequencies are minimised, and the dominant cell populations' frequencies are maximised. The range of D-score cutoffs where one of the dominant populations is absent are not considered. This determined cutoff is then applied to the D-scores of each cluster per marker, so that the clusters with a D-score above the threshold will be considered positive for that marker.

By default, the D-score cutoff is determined based on the co-expression patterns of the three T cell markers, CD3, CD4 and CD8a, to determine the positivity of all markers in the panel. We determine an optimal D-score cutoff that is applied for all markers to determine those clusters positive for the marker. If these three markers are present in the user's antibody panel, the optimal D-score cutoff will be determined based on these markers individually for each dataset.

The *typing params* config file specifies by default that the double-positive $CD4^{+}CD8a^{+}$ and single-positive $CD8a^{+}$ cells, for example, are expected to rarely be found in peripheral non-lymphoid tissue, whereas $CD3^{+}CD4^{+}$ and $CD3^{+}CD8a^{+}$ are the dominant populations in the analysed cohort. Based on this, TYPEx estimates an optimal D-score threshold that minimises the rare but maximises the dominant subpopulations (**Fig. 3c-d**). Overestimating the D-score cutoff results in a higher proportion of single-positive CD8a+ cells and overall Unassigned calls, whereas underestimating the D-score cutoff results in a higher proportion of double-positive $CD4^{+}CD8a^{+}$ T cells and overall Ambiguous calls (**Fig. 3c**). Two optimal thresholds are determined individually for the low and high-confidence cells in a cohort and applied across all clusters. These thresholds are estimated individually and automatically for each dataset, antibody panel and cohort. Therefore, even though only three markers are used to determine the D-score cutoff, this does not restrict the identification of other cell subpopulations in the dataset. Any three markers for which we can define high and rare frequency cell populations can be provided in the input config file (*typing params*).

An individual D-score threshold is determined for the low and high confidence groups, each of which has their own background distribution. Marker detection with TYPEx relies on the assumption that each marker intensity on a single cell represents one of two states, present/positive and absent/negative. This assumption is not fulfilled for proteins expressed on all or most of the measured cells. For example, except for some tumour cells, β2-microglobulin (B2M) is expected to be expressed on all nucleated cells. TYPEx aims to detect presence or absence of a protein, therefore intermediate states of lower expression may not

be identified as present. For example, this happens in cases of B2M when nearly all nucleated cells express the protein at different levels.

This approach does not assume that there is at least one marker positive in a given image or that all cells in an image need to be positive for at least one marker. We analysed all cells per antibody panel together, without considering or tracking which image the cells have been detected. Therefore, it does not assume that there is at least one marker positive within each image or that there is at least one or more markers positive within a confidence group, low or high.

**TYPEx: Automated cell type annotation**

Given a combination of expressed markers, a cell-type specificity score per cell type is calculated by weighting the frequency of those markers. For a given cell type *c* with $n_{defined_c}$ defining markers of which $n_{expressed_c}$are positive, TYPEx iterates through each marker *m* that defines cell type *c*, and weights the presence of each marker by the specificity of that marker (number of times it is found in other cell types). The sum of the weights is averaged by the number of markers. Therefore, the score will be the highest with a value of 1 for the most specific cell marker, which is defined as expressed only for one cell type (frequency=1) and no other cell marker will be necessary to define the corresponding cell type ($n_c$=1).

$$specificity\ score_c = \frac{n_{expressed_c}}{n_{defined_c}} * \sum_{marker\ m}^{defined_c} \left(\frac{expressed_m}{frequency_m}\right), expressed_m = \begin{cases} 1, detected \\ 0, otherwise \end{cases}$$

(2)

The required information for automated cell annotation is user-provided cell-type definitions and combinations of expressed markers. If provided, tissue segmentation and high-confidence major cell assignments will be considered as well. The assignment is generally independent of the stratification steps (*e.g.*, the probabilistic assignments) and considers the markers that are positive and define a cell subtype. The assigned cell subtype is determined as the one with the highest specificity score. When a cell is rendered Unassigned and it is located within the Tumour compartment based on tissue segmentation, this cell will be assigned to Epithelial/Tumour cells.

The low-confidence and high-confidence populations are both assigned based on the detected positive markers. However, in cases when the combination of expressed markers suggests an Ambiguous cell type call for a cell stratified as high confidence, the information from the probabilistic model will be considered. In such a case, the cell type annotation will be performed using the subtree of cell type definitions within the assigned major cell lineage.

This accounts for the cases when the detected positive markers on a given cluster were indicative of multiple cell types. For example, when a pancytokeratin signal from the neighbouring Epithelial cells is detected on a high-confidence CD8 T cell located within the tumour nests, we also considered the probabilistic cell assignment for the high-confidence CD8 T cell population (**Supplementary Fig. 5**). Alternatively, if in such a case this cluster is within the group of high-confidence Epithelial cells, it will be assigned to Epithelial cells. If this cluster was within the group of low-confidence Epithelial cells or CD8 T cells, this cluster will be assigned as Ambiguous.

Therefore, the number of clusters from the last stratification step does not equal the number of identified subpopulations. These are defined by the markers they express and the cell type annotations. For example, all clusters that are assigned Epithelial cells and express the same proteins will be considered the same cell subpopulation.

**Reproducibility analyses of the probabilistic models**

To evaluate how robust the probabilistic models are, we compared the major cell lineage assignments with CellAassign between three iterations of random subsampling (~2.1/3.16 million cells, **Supplementary Fig. 13a-b**). The CellAssign annotations reached a concordance of 91-95% among the subsampling runs.

**Impact of noise on cell phenotyping**

Different noise levels on marker channels from the T cell & Stroma panel in the TRACERx IMC dataset were simulated. Ion shot noise was simulated by modelling the ion counts as a Poisson process for a given channel, similar to previous work on noise simulation approaches on IMC data [7]. The pixel intensity with simulated noise was derived as:

$$S_p = P(\frac{R_p}{\gamma}), \{ \frac{\gamma = 1, high\ noise\ level}{\gamma = 2, low\ noise\ level} \} \tag{3}$$

where $S_p$ represents the intensities of the pixels p with simulated noise for a given channel, and P(r) is the Poisson noise. $R_p$ corresponds to the raw measured signal for the channel, and γ is the scale factor for adjusting the level of signal-to-noise ratio (SNR). Two noise levels were added to the raw signal: high noise level/low SNR where γ=1 and low noise level for γ=2. The Poisson noise was added to selected channels using the Python package numpy and its function random_noise with poisson distribution. Hot pixel removal and spillover compensation was performed as a pre-processing step before calculating the pixel intensities; therefore, hot pixel noise and signal spillover were not simulated in addition to ion shot noise.

We explored two scenarios:

1) if the noise levels of the threshold-defining markers (CD3, CD4, CD8a) remain constant and the noise levels of other markers change, how does the abundance of the other markers/cell populations change?

and

2) if the noise levels of the threshold-defining markers change, how does the abundance of all markers/cell populations change?

We first assessed, if the noise levels of the threshold-defining markers (CD3, CD4, CD8a) remain constant and the noise levels of other markers change, how did the abundance of the other markers/cell populations change. We considered three simulation scenarios: low noise levels for one of the markers (CD8a low), high noise levels for the same marker (CD8a high), and high noise levels for all three markers (CD3, CD4, CD8a). For each case, we evaluated the thresholds for the low and high confidence groups given the intensities of the defining markers with simulated noise. **Supplementary Figure S7a** shows the changes in the thresholds depending on the noise level and number of defining markers with simulated noise. Adding a low noise level to one of the markers resulted in similar thresholds within two decimal points for the raw and simulated data: 0 and 0.006 for high and low confidence groups, respectively. Adding high noise levels to one the three markers decreased the low confidence threshold by 0.045. Furthermore, high noise levels for all three markers decreased both the low and high confidence thresholds by 0.025 and 0.028, respectively. Therefore, these results demonstrated that the threshold estimate is affected by the noise levels of the defining markers.

We next evaluated the change in cell abundance as a result of the threshold variation (**Supplementary Figure S7b**). As a result of the threshold change with the added high noise level to CD8a intensities, 2.4% of the cells changed their cell type annotation. High noise levels for all three markers led to 6% of cells changing their annotation. Overall, we observed a strong correlation of the cell count for all cell types per image in pairwise comparison of the three runs with and without simulations. The minimum Spearman correlation coefficient from the pairwise comparisons was 0.96. In summary, noise and low signal-to-noise ratio can affect the performance of cell phenotyping on the corresponding channels, resulting in reannotation of up to 6% of cells. This observation highlights the need for evaluating the effect of noise systematically by cell phenotyping approaches for imaging mass cytometry data.

We next quantified the changes of respective cell marker positivity calls, when we imputed noise to the channels CD31 and αSMA, which were not used to define the D-score thresholds (**Supplementary Fig. 14c**). We compared the positive cells for the two markers: CD31 and

aSMA cells in the T cells & Stroma panel (**Supplementary Figure S7c-d**). The proportion of CD31+ cells with different noise simulation levels remained comparable to proportion based on the raw intensities, with an increase of up to 2.4% at high noise levels. Adding high noise levels to CD31 increased the proportion of Endothelial cells by 1.5% compared to the number of Endothelial cells based on raw CD31 intensities. However, high noise levels resulted in a decrease of 10% in cells positive for aSMA+ cells. Similarly, the number of cells annotated with the cell subtype aSMA$^+$ cells decreased by 4.6%. Of note, the change in cell subtype annotations is lower than the change in positive cells, because the cell assignment considers the combination of all positive markers. Finally, we compared pairwise the number of Endothelial cells and aSMA$^+$ cells per image between the three measurements no sim, low and high noise levels and observed a strong positive correlation (Spearman correlation coefficient $\rho=1$, $p<2e-16$).

**Cell phenotyping of rare cell populations**

Rare cell types, such as CD11c DCs and NK cells in the CRC CODEX dataset, showed poor correlation when comparing TYPEx cell type annotations as well as the manual annotations by Schürch *et al.* to the cell types derived from manual gating in the study by Schürch *et al.*[8]. These discrepancies can be attributed in part to the fact that markers for epithelial cells (pancytokeratin) and αSMA$^+$ cells were not analysed with the manual gating approach. Therefore, there were many CD11c DCs based on manual gating that were instead annotated as epithelial cells in the TYPEx annotations and the manually curated cell subtype annotations in the study by Schürch *et al*.

To demonstrate that the performance of TYPEx is not limited by the frequency of the cell population, we used published ground truth annotations derived from iterative clustering and manual curation using the healthy intestine tissue from the HuBMAP dataset[9]. Mean recall and precision are shown in **Supplementary Fig 13e**. The rarest cell populations had lower F1-scores relative to the mean F1-score: Paneth (n=216), Enterocyte_ITLN1p (n=1558) and Neuroendocrine (n=1623). However, higher F1-scores were observed for cell subtypes with similar frequency in the cohort: Neutrophil (n=1983), ICC (n=2140), B (n=2202) and DC (n=2273). The cell subtypes represented by at least ~6000 cells generally had F1-scores close to or above the macro F1-score, except for Lymphatics (n~6000). These results suggest that, while the performance of TYPEx may be suboptimal for some rare cell subpopulations, likely due to lower specificity of the defining markers, TYPEx can capture both rare and frequent subpopulations with comparable accuracy. The corresponding confusion matrices for Schürch *et al.*, BE and the HuMAP dataset are included in **Supplementary Fig. 11b, Supplementary Fig. 12a** and

**Supplementary Fig. 12c**, respectively. The accuracy metrics are included in **Supplementary Fig. 13c-e**.

### Barrier score validation

We simulated 1,448,979 cells across 228 1000x1000 pixel images for a model tumour with a simplified circular structure and an αSMA⁺ fibroblast barrier at the edge, maintaining realistic densities of other cell types intratumourally and within the stroma in line with measured values across our experiments (**Supplementary Fig. 7c-e**). These densities were taken from measured values for the TRACERx 100 IMC cohort images. We applied the Spatial-PHLEX workflow to these images with DBSCAN *EPS*=35 and showed that higher barrier scores to tumour cell clusters reflect both an increased fraction of barrier cells in the adjacent-to-tumour region, and an increased barrier thickness.

### Impact of segmentation approaches on cell phenotyping and barrier score

To examine how segmentation impacts the detection of cell phenotypes, we performed cell segmentation with dilation of 1px, 5px and 10px, using deep-imcyto in simple mode for panel T cells & Stroma. Each of the segmentation outputs were then used as input to TYPEx for cell type analysis. The cell phenotyping results from the three dilation runs were then compared to those derived from the Multiplexed Consensus Cell Segmentation (MCCS) approach (**Supplementary Fig. 15a-f**).

We observed strong positive correlation for all major cell types identified with the panel T cells & Stroma, except for T cells - Other (n=102 cells with MCCS) (**Supplementary Figure S8a**). However, increasing the dilation pixels resulted in a higher proportion of double-positive $CD4^+CD8a^+$ T cells and higher count of cells rendered Ambiguous (**Supplementary Figure S8b-c**). The proportion of double-positive T cells was twice higher in dilation runs with 5 and 10 pixels compared to 1 pixel and MCCS. Furthermore, as the number of dilation pixels increased, the number of identified Endothelial cells decreased. Similarly, the highest number of aSMA+ cells and Endothelial cells were captured with the MCCS approach compared to the dilation approaches. These results confirmed that MCCS segmentation results in higher stromal content and higher number of typed $aSMA^+$ cells and Endothelial cells compared to simple segmentation approaches, with 1-, 5-, and 10-pixel dilations.

To evaluate the detection of Ambiguous cells with increased signal spillover from neighbouring cells, we ran TYPEx on cell objects segmented in *simple* mode of deep-imcyto with 1-pixel dilation and 5-pixel dilation using the test dataset (**Supplementary Fig. 17, Fig. 4a**). Increasing the neighbour signal with 5-pixel dilation around the nuclear objects resulted with

an increased number of Ambiguous calls detected by TYPEx in the areas with dense immune cell distribution compared to 1-pixel dilation and the MCCS procedure in *CellProfiler* mode.

We also examined how ɑSMA$^+$ fibroblast barrier scores varied when either including or excluding the non-nucleated cells picked up during the MCCS procedure as a means of assessing the impact on conclusions drawn by incorporating these additional cells using our workflow (**Supplementary Fig. 15g-h**) shows a Spearman correlation plot for the ɑSMA$^+$ fibroblast barrier scores calculated across 121 LUAD and LUSC tumour cores from the TRACERx lung dataset when including all cells ('All Cells') or excluding the non-nucleated cells identified using MCCS ('Nucleated Cells Only'). Whilst individual cores show changes in the scores using the different segmentation approaches, the overall Spearman correlation is very high ($\rho$=0.95, p<2.2e-16), suggesting that, in this case, the segmentation approach may be unlikely to change overall conclusions related to barrier score analyses. Nonetheless, individual regions of interest provide compelling examples as to the benefits of including non-nucleated aSMA+ cells in better recapitulating tissue structure (**Supplementary Fig. 15h**). In the bottom right panel, non-nucleated cells are represented in white, providing an example of an instance in which peritumoural aSMA$^+$ cells would not be picked up by an approach relying solely on nuclear seeds.

# Supplementary Figures

a

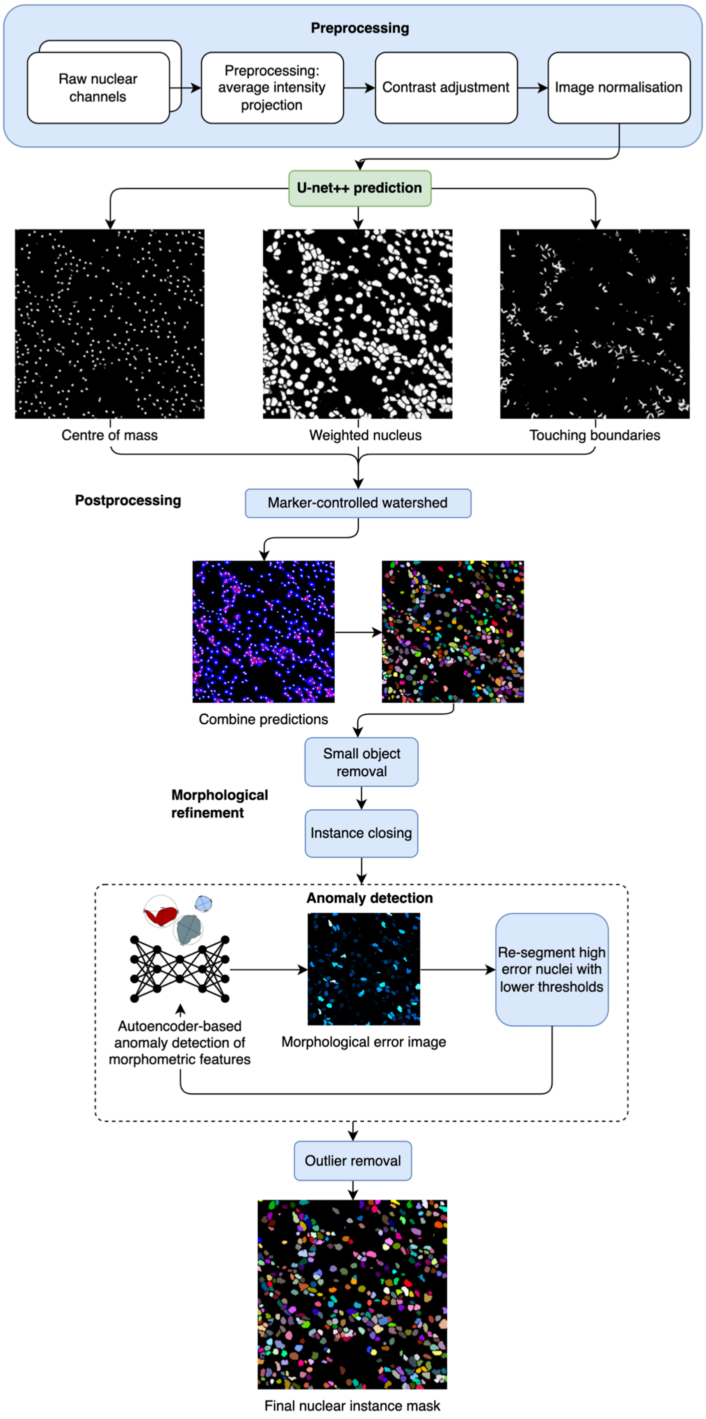


b

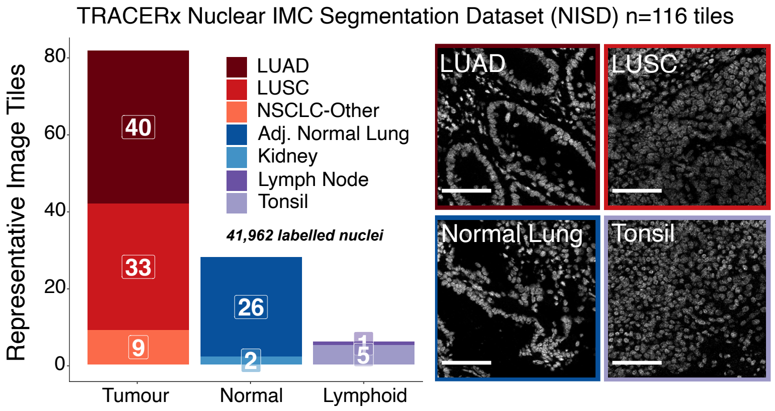


**Supplementary Figure 1**

**Supplementary Figure 1: Nuclear segmentation procedure implemented in deep-imcyto.**

**a,** IMC channels containing nuclear stain information as specified by the user in the deep-imcyto configuration are preprocessed and normalised for input into the deep-imcyto U-net++ prediction model, which independently predicts multiple features of the input image pertaining to the presence of nucleus-like objects: centre of mass, weighted nucleus, and touching boundaries. These semantic predictions are combined via marker-controlled watershed into a label image representing all unique nuclei. These labels are then postprocessed to improve the accuracy via morphological processing techniques: (1) small object removal (<4px) and (2) a procedure we term “instance closing”, whereby each nuclear object in the label image has a binary closing procedure performed on it as if it were a binary object in an otherwise empty image. This has the effect of removing internal cracks within each object, without modifying inter-nuclear spaces between each object. Uniquely after this step we use deep anomaly detection based on an autoencoder model trained to identify nuclei with improbable morphometric parameters. We use the autoencoder to flag nuclei that have been segmented poorly, then re-segment the underlying image area with changed watershed parameters, before applying the autoencoder again to detect any remaining morphometry outliers. We then remove these remaining outliers from the final nuclear label image (*i.e.* instance mask). Qualitatively we observed that two rounds of outlier removal were sufficient to improve the poorly segmented nuclei in most images. **b,** Breakdown of lung cancer, tumour-adjacent normal, lymphoid and kidney tissues within the nuclear annotation dataset used to train deep-imcyto, the TRACERx Nuclear IMC Segmentation Dataset (NISD, n=41,962 nuclei). Example training tiles exhibit the morphological variety of nuclei and tissue type composition of the dataset. Source data are provided as a Source Data file. Scale bar=80µm. IMC, imaging mass cytometry; LUAD, lung adenocarcinoma; LUSC, lung squamous cell carcinoma, NSCLC, non-small cell lung cancer.

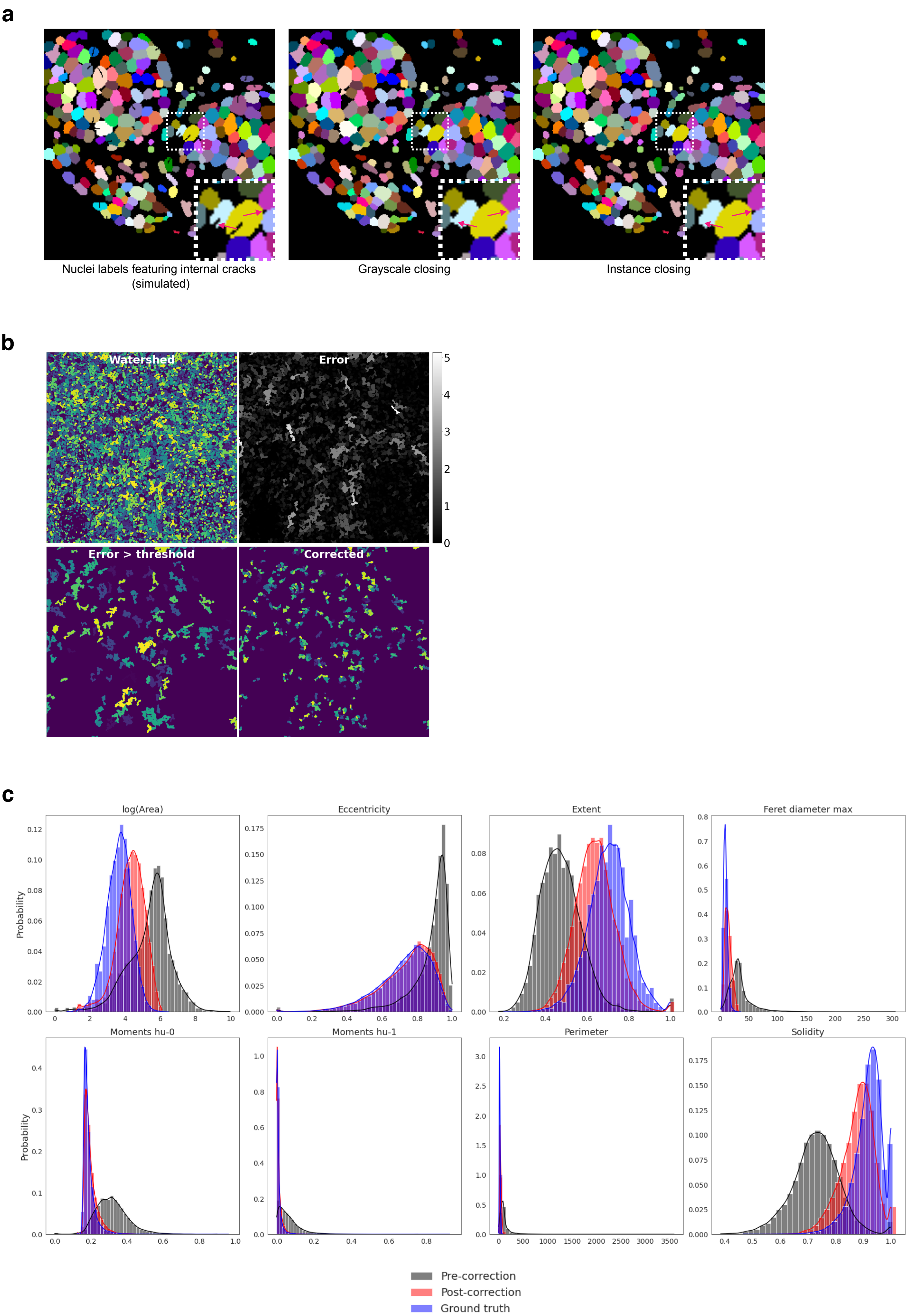


**Supplementary Figure 2**

**Supplementary Figure 2: Effects of the deep-imcyto post-processing procedure.**

**a,** The effect of morphological instance closing vs grayscale closing for healing internal artefacts in nucleus labels, showing that instance closing preserves the perimeter of the label, where grayscale closing does not. Crack artefacts were artificially introduced to a nucleus mask from the TissueNet dataset. **b,** Panels show the process of autoencoder prediction refinement in a challenging imaging mass cytometry (IMC) region for segmentation. (Top left) The initial watershed produced from UNet++ initial predictions. Multiple large irregular labels are apparent, resulting from merged nuclei. (Top right) The autoencoder error associated with each label shows higher values for irregular regions. (Bottom left) Label regions above the chosen threshold are all irregular and thus candidates for re-segmentation. (Bottom right) Re-segmenting these regions through watershed with looser threshold parameters on the UNet++ probability map improves the segmentation of nuclei within these regions. These are then reincorporated into the original label image in place of the high-error labels. **c,** Quantitative improvement of the measured distribution of eight morphometric parameters relative to the ground truth morphometry (measured from the TRACERx Nuclear IMC Segmentation Dataset) in regions that were flagged via autoencoder and re-segmented.

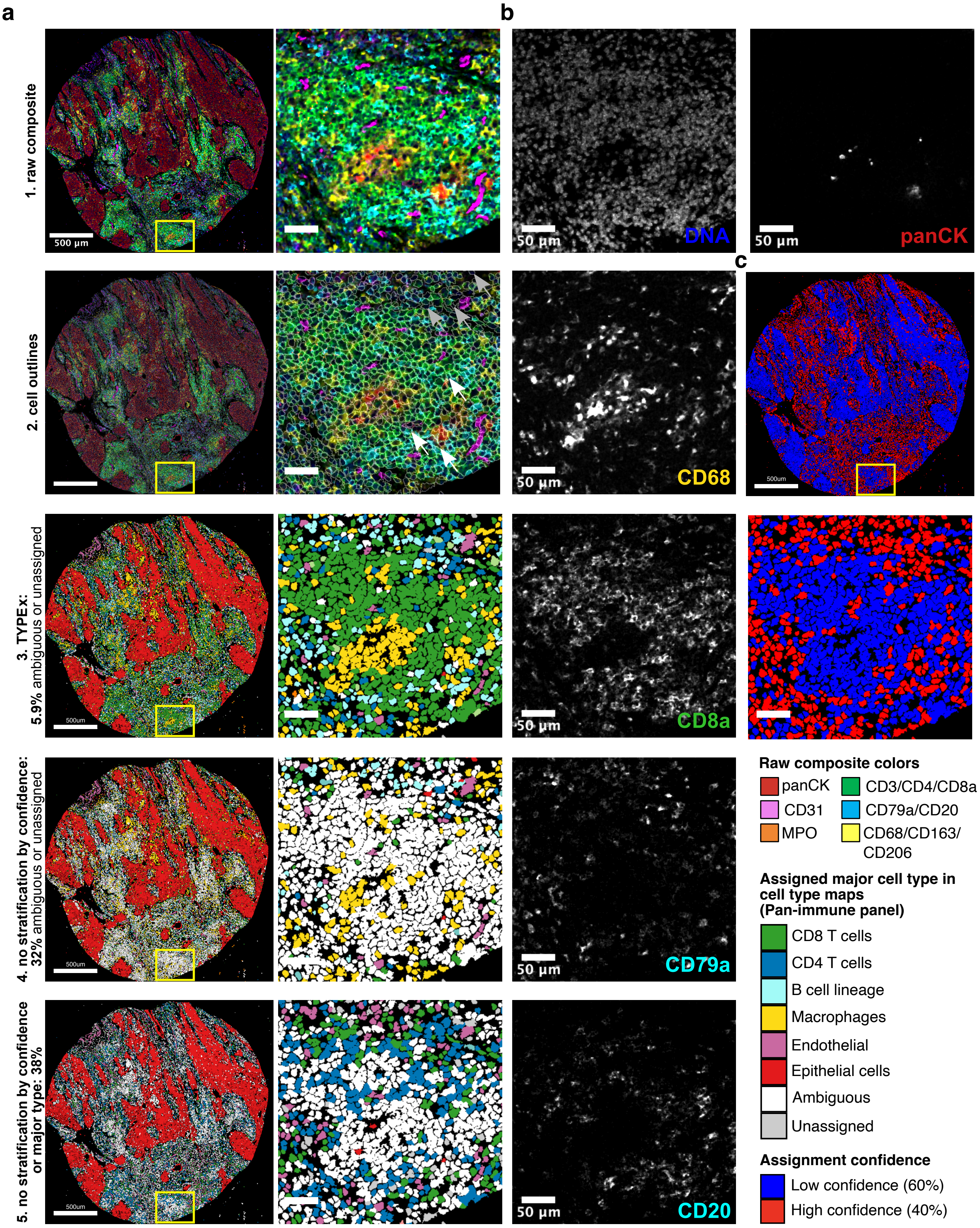


**Supplementary Figure 3**

**Supplementary Figure 3: The impact of the different stratification steps in TYPEx on cell phenotyping demonstrated on an example image from the TRACERx 100 IMC cohort.**

**a-b,** For a case with 60% low confidence major cell type calls in the cell stratification stage of TYPEx, several images of raw marker intensities, cell object outlines and cell types are shown. In **(a)**, a composite image illustrates the pseudo-coloured intensities of major cell type-specific markers **(1)**. The colours and the represented markers are indicated in the composite colour legend. An aggregate of immune cells is shown in the zoomed area highlighted in yellow (333µm x 333µm). Cell outlines are shown for segmented cells **(2)**, with white and grey arrows indicating examples of ambiguous and unassigned cells, respectively, which remain so after the full TYPEx workflow. In the cell stratification stage, TYPEx stratifies the cells first by putative major cell lineage, second by confidence, and third by cell clustering. The three stratification steps in TYPEx led to 5.9% of cells assigned as ambiguous or unassigned **(3)**, whereas excluding stratification by confidence resulted in 32% ambiguous or unassigned cell assignments **(4)**. Further excluding stratification by major cell lineage, *i.e*. solely performing clustering, resulted in 38% cells with ambiguous or unassigned annotations **(5)**. **b,** Raw intensity images from single channels representing cell type-specific markers for the assigned cell types in the zoomed area of **(a)**. **c,** Low and high confidence major cell type calls identified with the TYPEx stratification-by-confidence step are highlighted in blue and red, respectively.

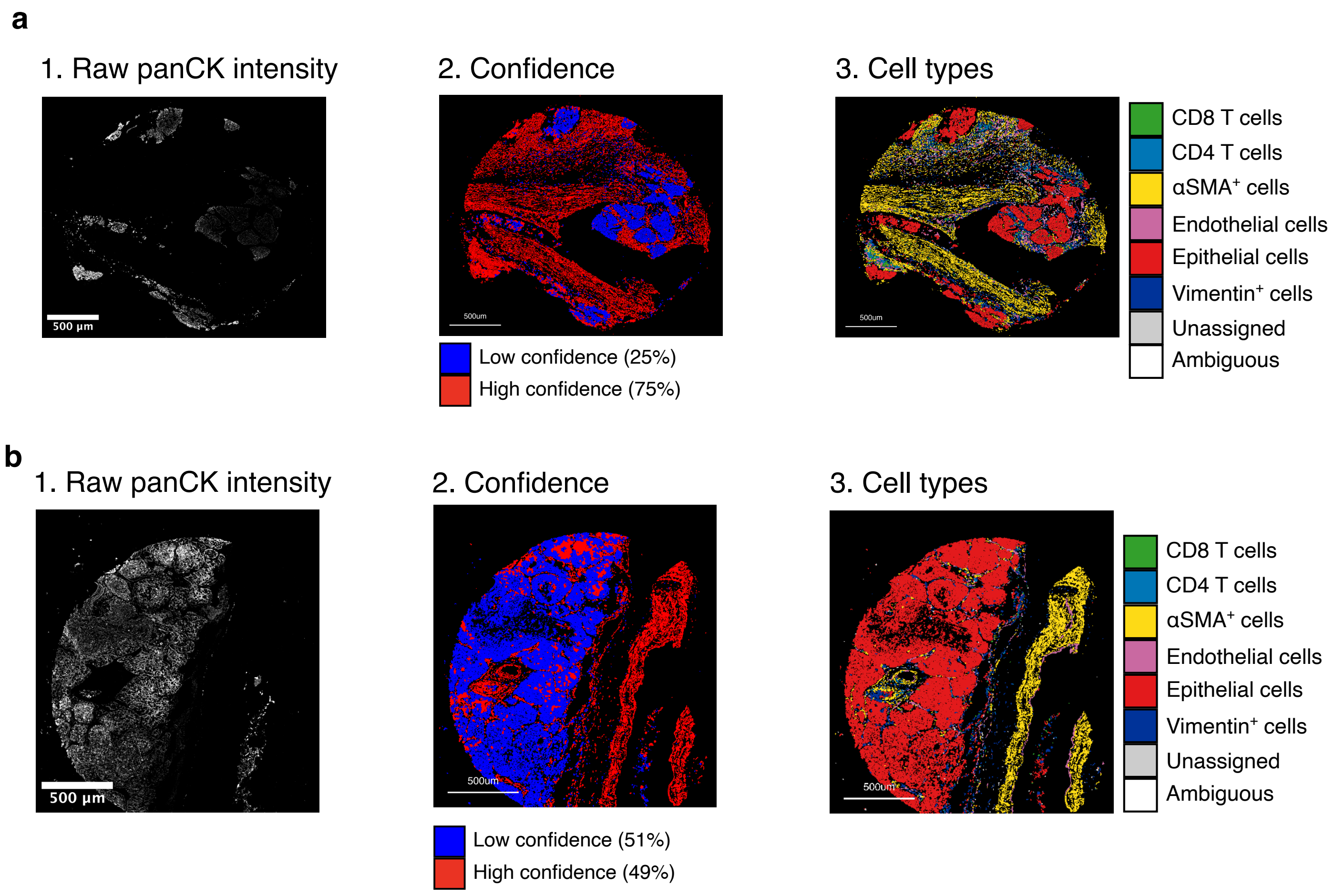


**Supplementary Figure 4**

**Supplementary Figure 4: Examples of images with low and high confidence cells.**

**a,b,** Each example illustrates variable pancytokeratin intensities within the imaged tissue area and their assigned confidence groups. **(1)** shows the raw pancytokeratin (panCK) intensity image. **(2)** visualises the cells in an image coloured based on their low or high confidence group. The typed cell identities are visualised in **(3)**.

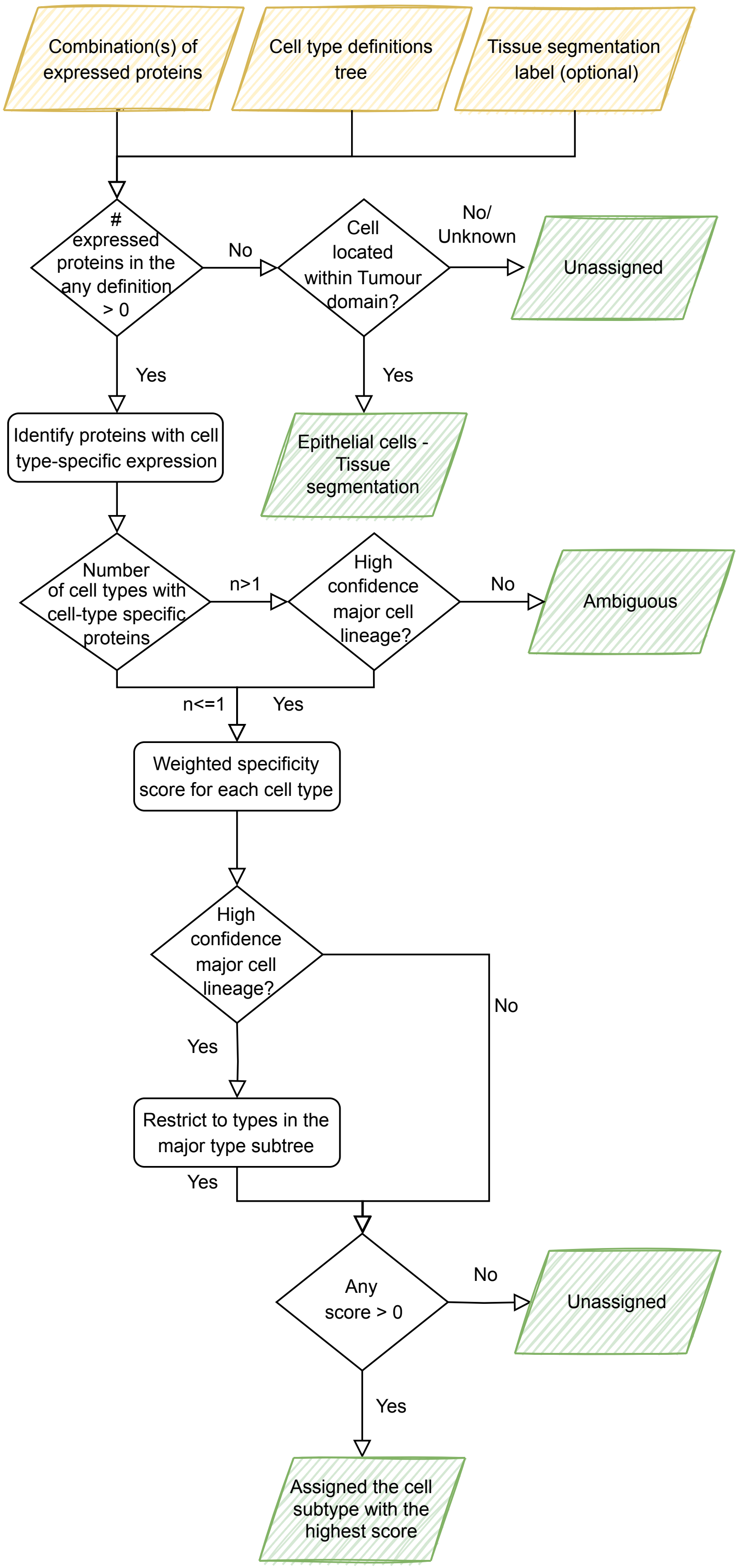


## Supplementary Figure 5

**Supplementary Figure 5: A flowchart for automated cell assignment given a combination of expressed protein markers.**

The required input for automated cell annotation is user-provided cell type definitions and combinations of positive markers. If provided, tissue segmentation and high-confidence major cell lineage assignments will be considered as well. Markers with cell-type specific expression are those defined for only one cell subtype in the cell type definitions file. To identify the best cell type match for a combination of markers, a weighted specificity score is calculated based on the average frequency of the positive markers in the definitions tree and the number of profiled markers a cell type can express. The assignment is generally independent of the stratification steps. When a cell is rendered Unassigned and it is located within the Epithelial/Tumour compartment based on tissue segmentation, this cell will be assigned to Epithelial/Tumour cells. In cases when the combination of expressed markers suggests an Ambiguous cell type call for a cell stratified as high confidence, high-confidence information from the probabilistic model will be considered. In such a case, the cell type annotation will be performed using the subtree of cell type definitions within the high-confidence major cell lineage.

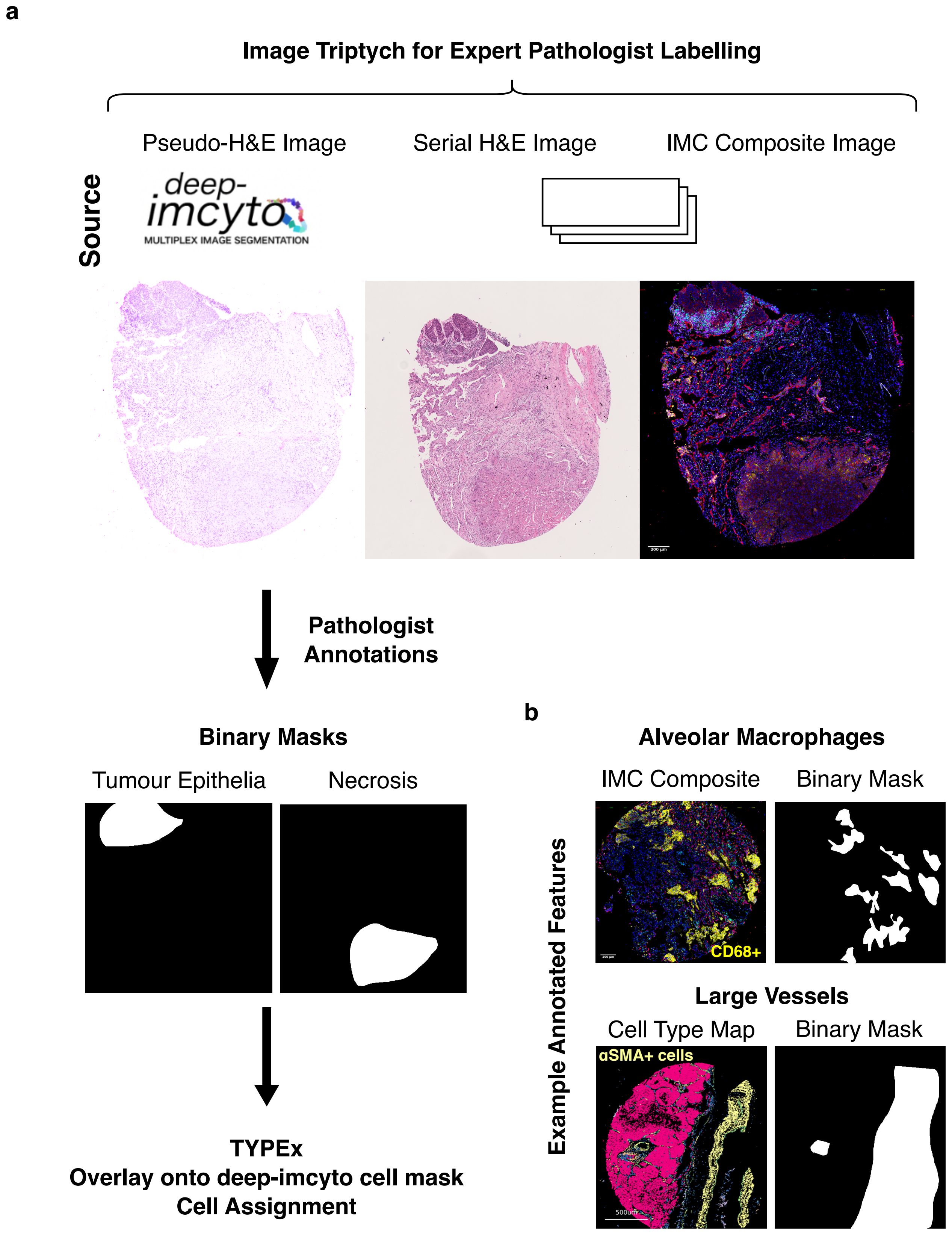


**Supplementary Figure 6**

**Supplementary Figure 6: Defining binary tissue masks to which cells can be assigned using pathologist annotations.**

**a,** Pathologist tissue annotation workflow. Pseudo-H&E, H&E and IMC pseudo-coloured composite images are combined into a single file compatible with manual annotation software, such as NDP.view2. Manual annotations such as tumour epithelia and necrosis are converted into binary masks, which are then overlaid onto the cell mask output from deep-imcyto for detailed localisation of TYPEx cell types. **b,** Binary masks of example pathologist annotated features, Alveolar Macrophages and Large Vessels, are shown with accompanying pseudo-coloured composite IMC image with the marker CD68 (Alveolar Macrophages) and cell type map with αSMA (Large Vessels) represented in yellow, respectively. H&E, haematoxylin and eosin; IMC, imaging mass cytometry.

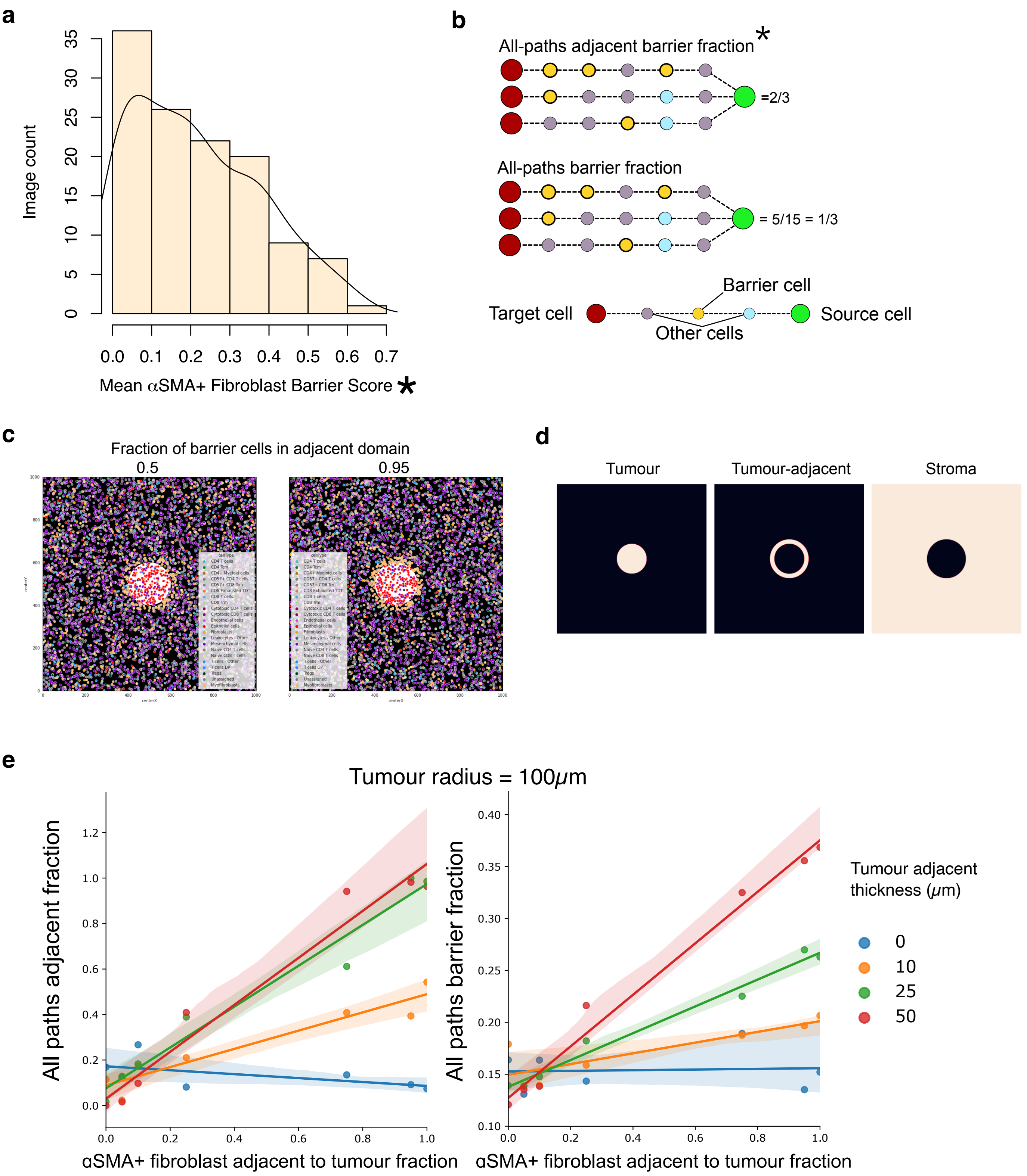


**Supplementary Figure 7**

**Supplementary Figure 7: Spatial-PHLEX cellular barrier quantification and validation with simulated cell position data.**

**a**, Average barrier score for CD8 T cell access to tumour epithelial cells across 133 images. * indicates that the barrier score used is the all-paths adjacent barrier fraction indicated by a * in (**b**). **b**, Visual representation of barrier metrics derived by Spatial-PHLEX. **c,** Example scatter plots of simulated positional data for a 100µm radius tumour with a varying fraction of barrier cells in the tumour-adjacent domain. Different colours represent different cell types on a black background. **d**, Domain definitions used in barrier simulations with selected features indicated in beige. **e**, Score results of multiple simulations of a 100µm radius cluster of epithelial cells with varying tumour-adjacent domain thicknesses, surrounded and infiltrated by immune/stromal cells. Results for the all-paths adjacent fraction barrier score (left). Results for the all-paths barrier fraction score (right). Cell densities in Stroma and Tumour tissue compartments were simulated to reflect measured values from the TRACERx 100 imaging mass cytometry (IMC) cohort. $\alpha SMA^+$ fibroblast density is varied in the tumour-adjacent domain. In general, higher proportions of $\alpha SMA^+$ fibroblasts at the tumour-stroma boundary results in higher barrier scores, as does a greater tumour-adjacent domain thickness. Source data are provided as a Source Data file.

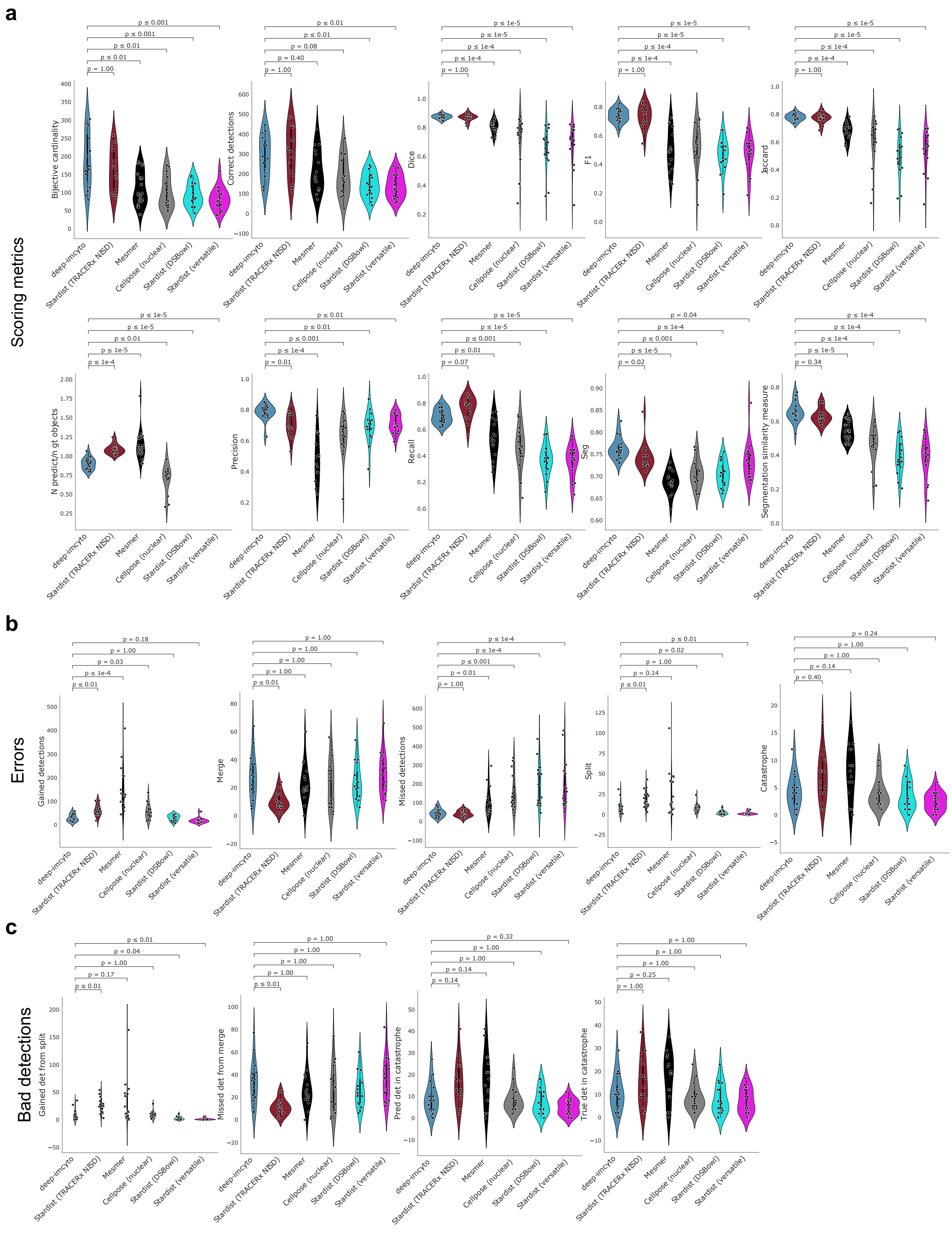


**Supplementary Figure 8**

**Supplementary Figure 8: Performance evaluation of deep-imcyto compared to other segmentation models.**

Performance of deep-imcyto nuclear segmentation *vs* other models evaluated through 19 performance metrics, split by class of metric. These consist of the 16 segmentation assessment metrics of the DeepCell toolbox plus the segmentation similarity and bijective cardinality metrics of Al-Kofahi *et al*., and are split into **a**, scoring metrics (higher is better), **b**, error metrics (lower is better), and **c**, bad detection metrics (lower is better). Other segmentation models included in the comparisons are the generalist cell segmentation models Mesmer and Cellpose, and two nuclear segmentation models, which are distributed with Stardist: the DataScienceBowl 2018 model and the versatile model. We also compared deep-imcyto against an imaging mass cytometry (IMC)-specific Stardist model, which we trained on the TRACERx Nuclear IMC Segmentation Dataset (NISD). Violin plots are shown, each dot represents the score of a given segmentation method for each image of the held-out test dataset. P-values represent the Mann-Whitney U-test.

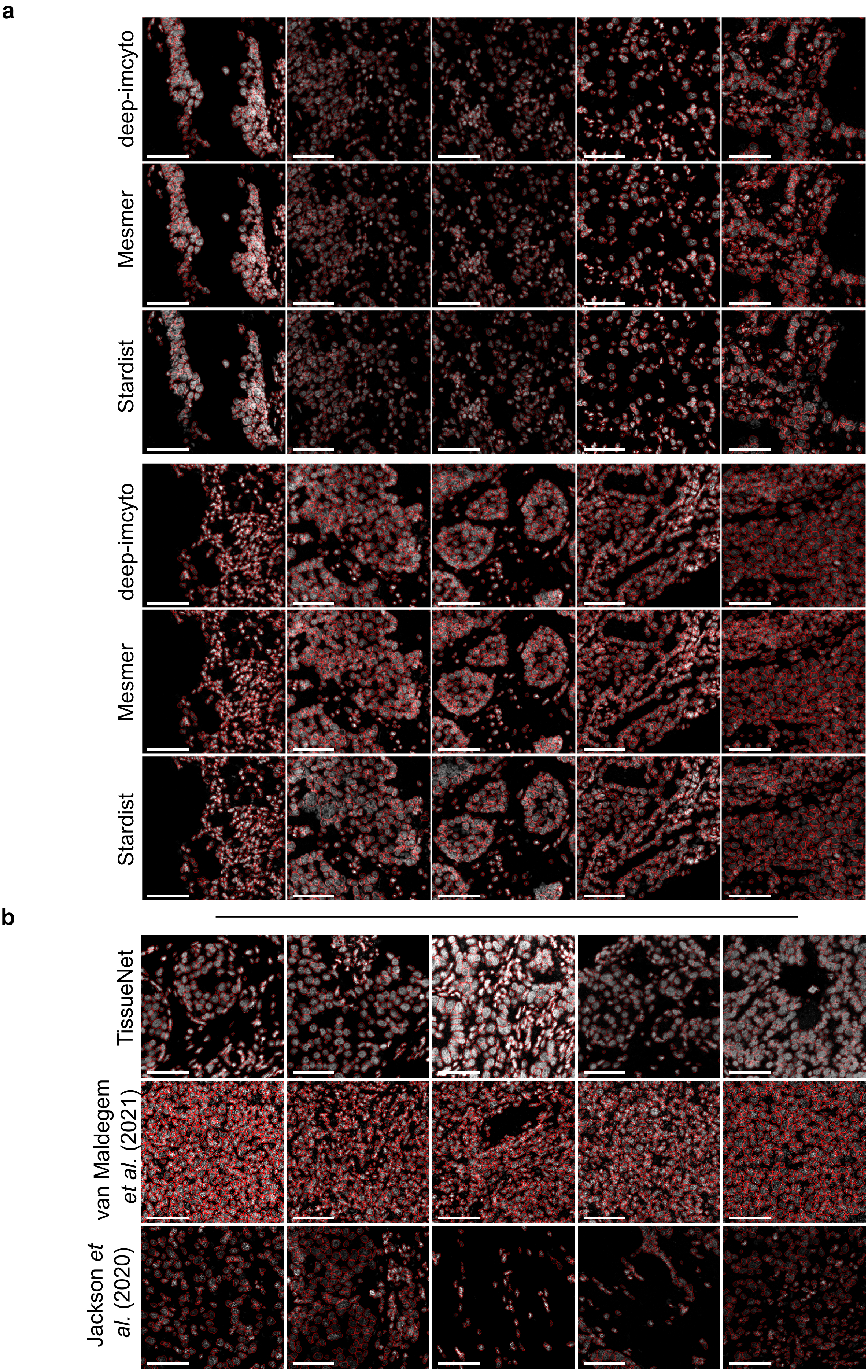


**Supplementary Figure 9**

**Supplementary Figure 9: Qualitative examples of deep-imcyto nuclear segmentation.**

**a**, Side-by-side comparison of deep-imcyto, Mesmer and Stardist nuclear segmentation on TRACERx 100 IMC lung cancer data. deep-imcyto captures underlying nuclear morphology across a range of tissue contexts, cell densities, and signal strengths. **b**, deep-imcyto nuclear segmentation performance on three publicly available IMC datasets encompassing different tissue types and preparation methods: TissueNet breast cancer data[10], frozen mouse lung cancer data from van Maldegem *et al.* (2021)[11], FFPE breast cancer data from Jackson *et al*. (2020)[12]. For both panels: DNA shown in grey, segmentation outlines in red, image crop size 256x256µm, scale bar=75µm. IMC, imaging mass cytometry.

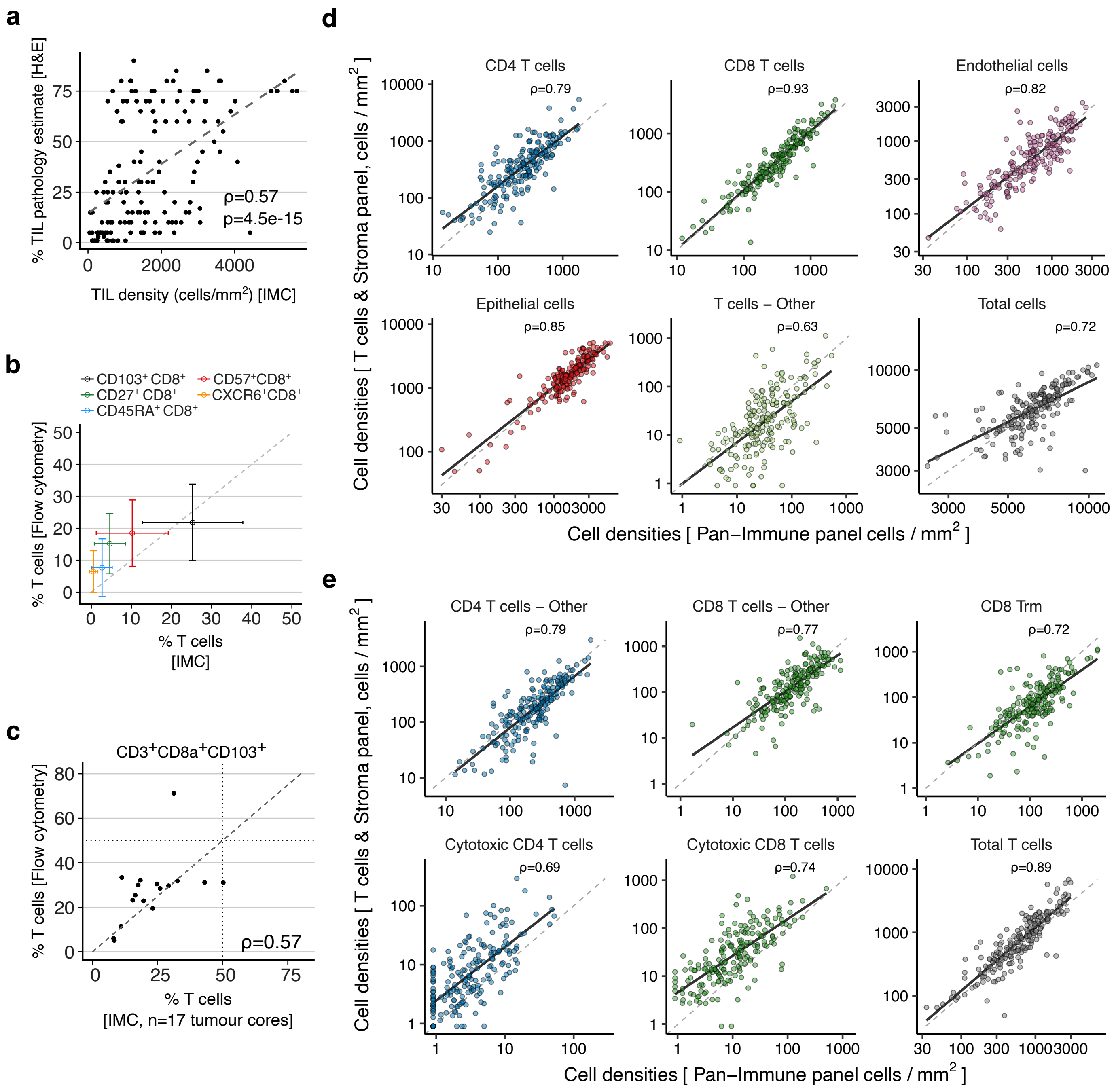


**Supplementary Figure 10**

**Supplementary Figure 10: TYPEx validation with orthogonal TRACERx data.**

**a-c,** Validation of TYPEx using orthogonal TRACERx data. Correlation between pathologist-labelled TIL scores from regional H&E images and the IMC-derived TIL density from paired TMA cores (n=157 tumour cores from 70 patients) **(a)**. TIL scores represented the area occupied by mononuclear inflammatory cells over the total viable intratumoral and stromal areas. The TIL densities were calculated as the T cells and B cells count per tissue area [$mm^2$]. Validation of TYPEx using orthogonal flow cytometry data in TRACERx (n=67 tumour regions, 30 patients) **(b)**. Each point indicates the mean and the error bars are the standard deviation of the T cell subpopulation frequencies. The frequency of $CD8^+$ tissue resident memory T cells ($CD3^+CD8a^+CD103^+$) was correlated between TYPEx and flow cytometry measurements, in a subset of 17 tumour regions with a comparable fraction of assayed T cells (Spearman correlation) **(c)**. **d-e,** Correlation of cell density between two IMC antibody panels applied on matched TMA cores in the TRACERx 100 IMC dataset. Pan-Immune panel and T cells & Stroma panel per major cell lineage **(d)** and cell subtype **(e)**. Source data are provided as a Source Data file. ρ, Spearman correlation coefficient; TIL, tumour infiltrating lymphocytes; H&E, haematoxylin and eosin; IMC, imaging mass cytometry; Trm, T resident memory.

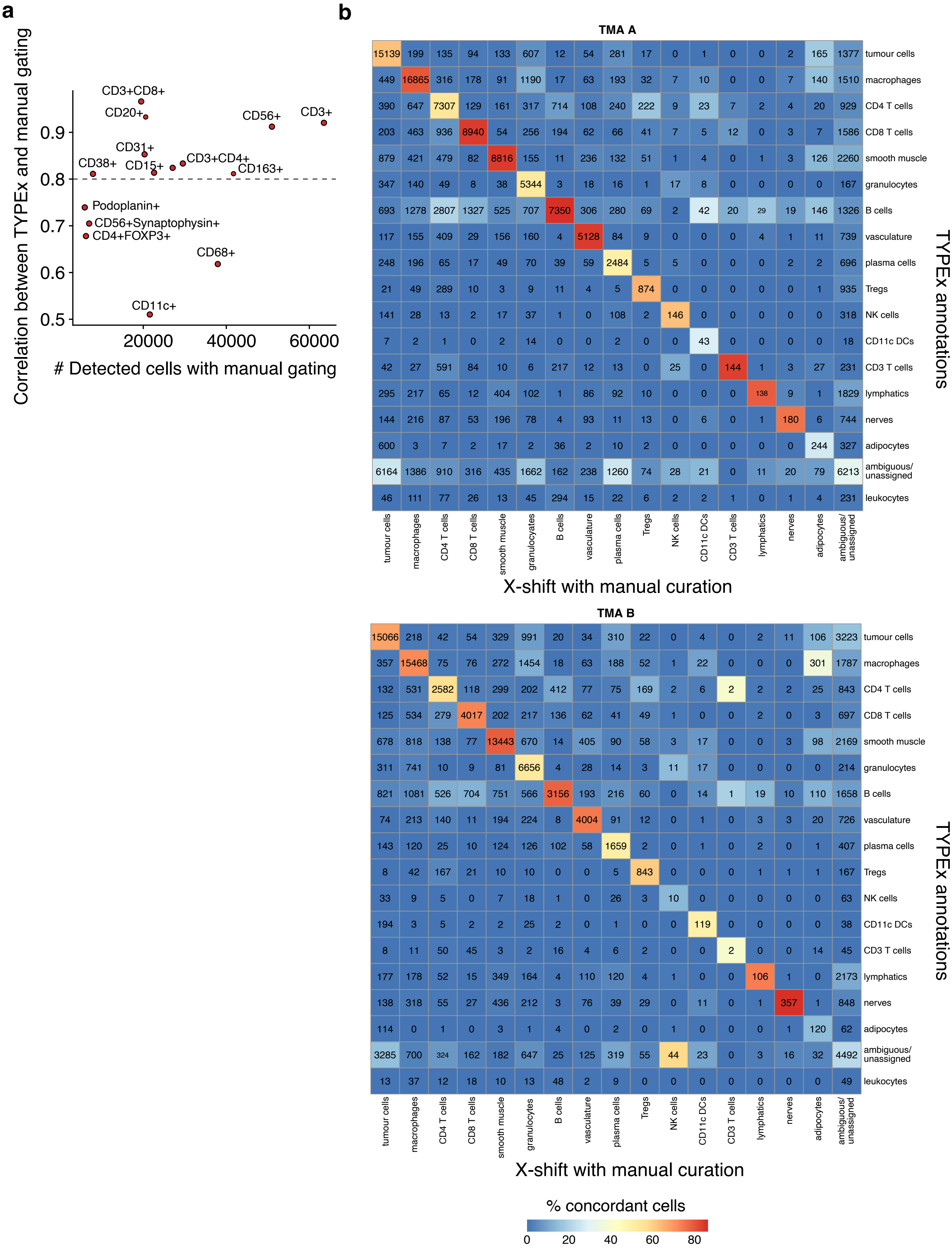


**Supplementary Figure 11**

**Supplementary Figure 11: TYPEx validation with public CODEX imaging data.**

**a,** Spearman correlation coefficients of marker-positive cell counts between TYPEx and manual gating on the colorectal cancer (CRC) CODEX data against the size of the cell populations from manual gating (n=140 cores, 35 patients). **b,** Confusion matrices comparing TYPEx to manually curated published annotations shown separately for the two tissue microarrays (TMAs), TMA A and TMA B, in the CRC CODEX dataset (n=70 cores per TMA, Schürch *et al.*). Numbers in squares represent cell count. Source data are provided as a Source Data file.

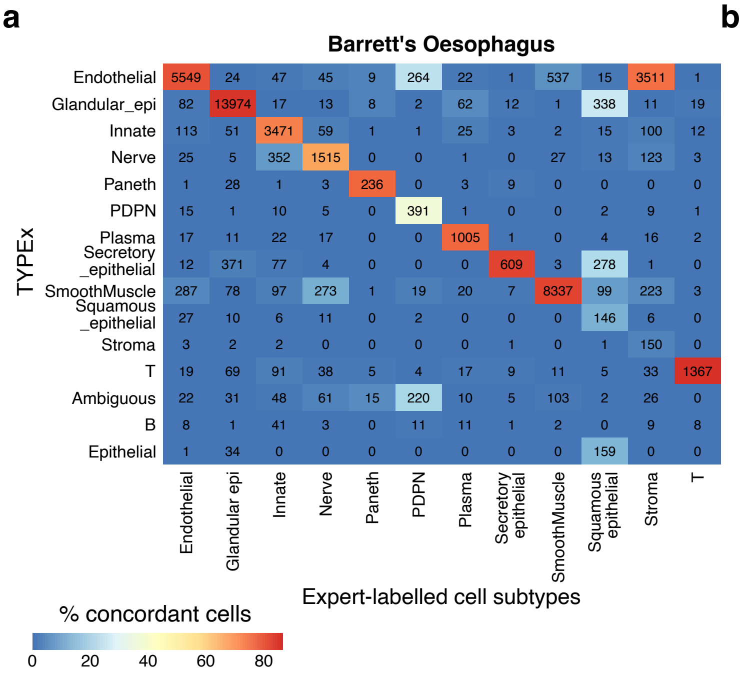


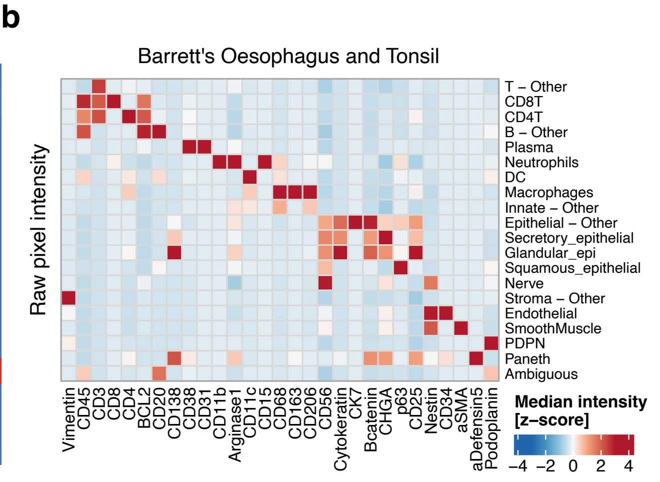


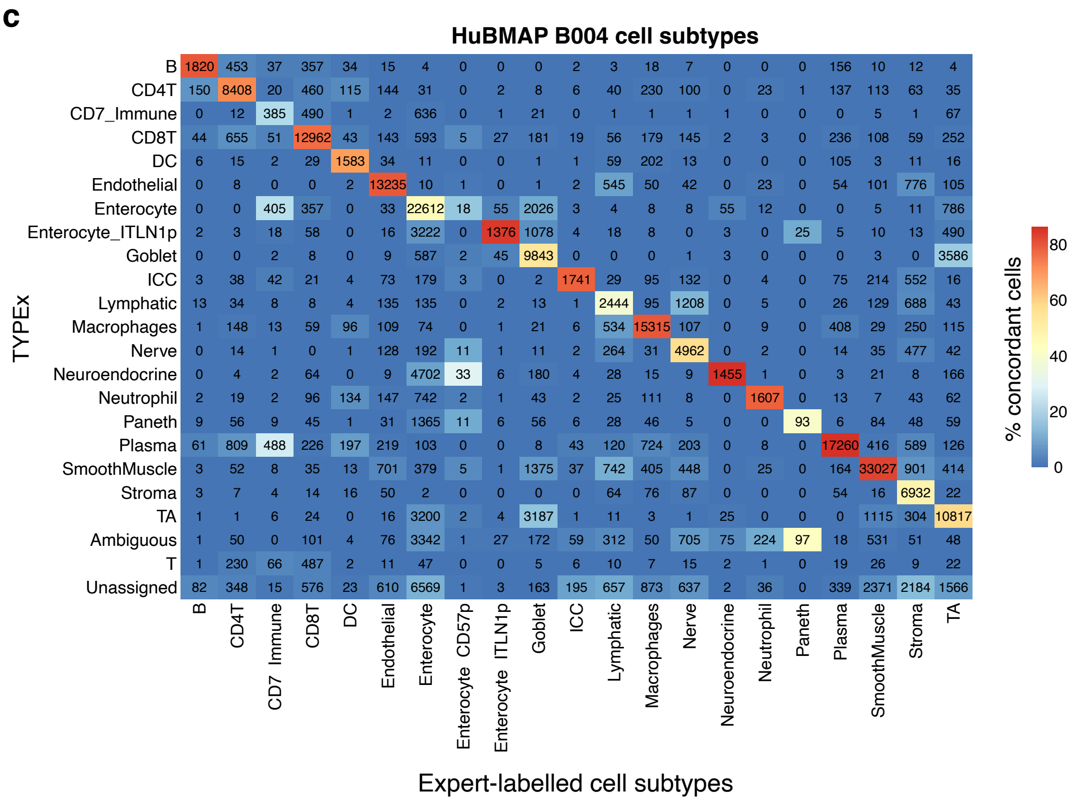


# Supplementary Figure 12

**Supplementary Figure 12: Benchmarking and validation of TYPEx using public CODEX imaging datasets.**

**a,** Confusion matrix of cell types identified by TYPEx (rows) and ground truth annotations (columns) for the Barrett's oesophagus (BE) dataset (n=1 whole slide image). **b,** Heatmap showing the distribution of z-score normalised median pixel intensities for markers across different cell subtypes annotated by TYPEx for the BE and tonsil datasets. Median pixel intensities are calculated as the median of cell-level mean pixel intensity values for the relevant marker and cell subtype. **c,** Confusion matrix comparing TYPEx cell annotations to expert-labelled cell subtypes on the tonsil dataset used to train STELLAR. The evaluation metrics were lower on the tonsil dataset used to train STELLAR, where 10% were Ambiguous calls, largely due to PDPN and CD20 co-expression**.** Source data are provided as a Source Data file.

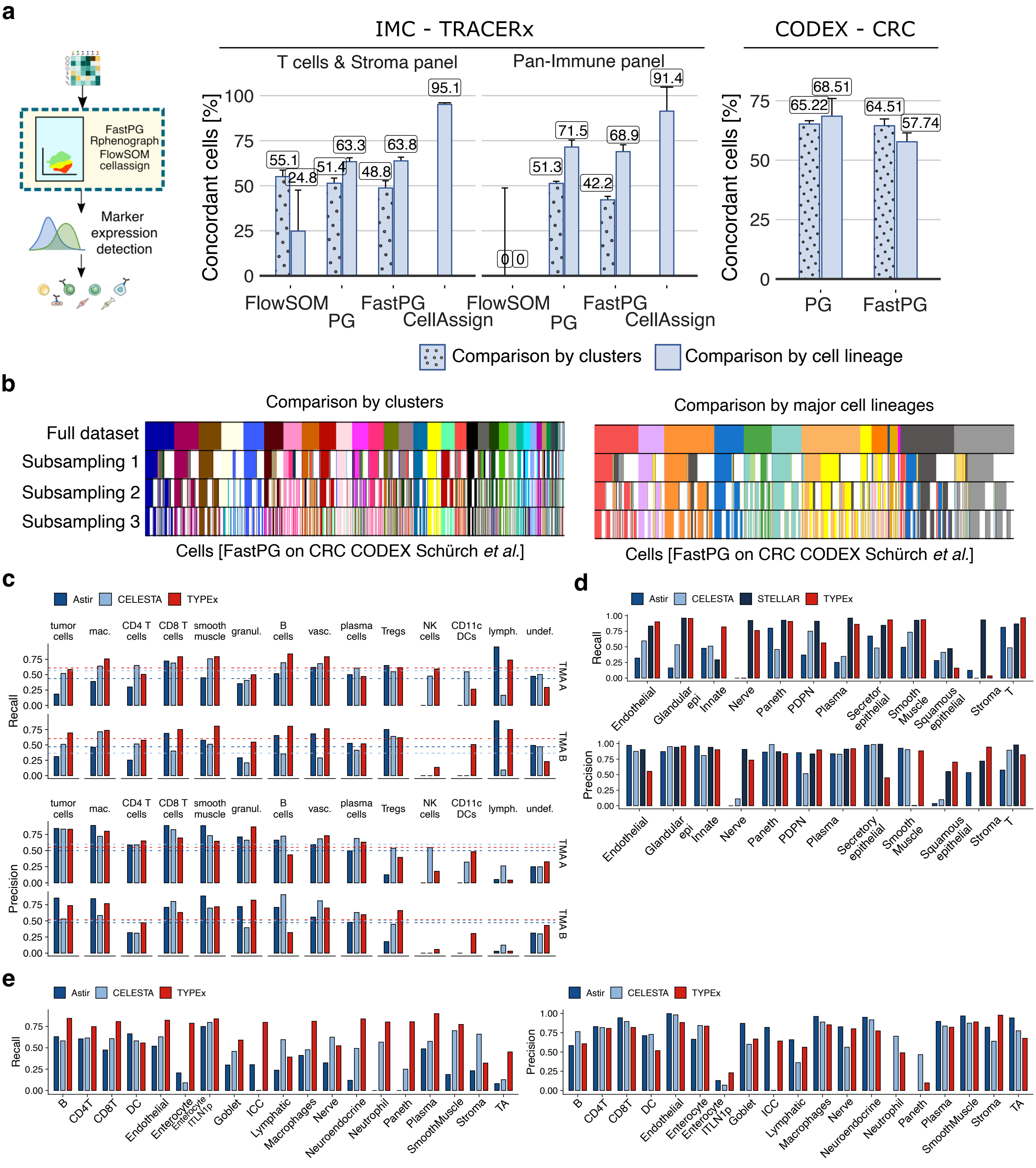


**Supplementary Figure 13**

**Supplementary Figure 13: Benchmarking of TYPEx against cell phenotyping approaches.**

**a,** Comparison of clustering approaches results with random ⅔ subsampling of cells. The clustering approaches, FlowSOM, FastPG and PhenoGraph (PG) and the supervised probabilistic method, CellAssign, were applied on TRACERx 100 IMC (left, n=2.1M out of 3.16M cells for both antibody panels) and the colorectal cancer (CRC) CODEX dataset (right, n=172,257 out of 258,385 cells). **b,** Comparison of cluster and major cell lineage results with FastPG clustering between subsampling runs on the CRC CODEX dataset. The full dataset was used as a reference for comparing the three ⅔ randomly sampled subsets (n=172,257 out of 258,385 cells). **c,** Recall (left) and precision (right) metrics for the cell subtypes identified by TYPEx, CELESTA, and Astir against the expert-labelled annotations in the CRC CODEX dataset. CELESTA metrics for TMA A were derived from a published confusion matrix. Average recall or precision metrics for each typing method across all cell subtypes and TMAs are shown by coloured dashed lines. **d**, Performance evaluation metrics recall (left) and precision (right) for each cell subtype identified by TYPEx, CELESTA, Astir and STELLAR compared to ground truth annotations in the samples from fresh frozen tissue sections of Barrett's oesophagus (BE). **e,** Performance evaluation metrics recall (left) and precision (right) for TYPEx, CELESTA and Astir cell annotations for samples from patient B004 in the HuBMAP dataset. Source data are provided as a Source Data file. TMA, tissue microarray. IMC, imaging mass cytometry.

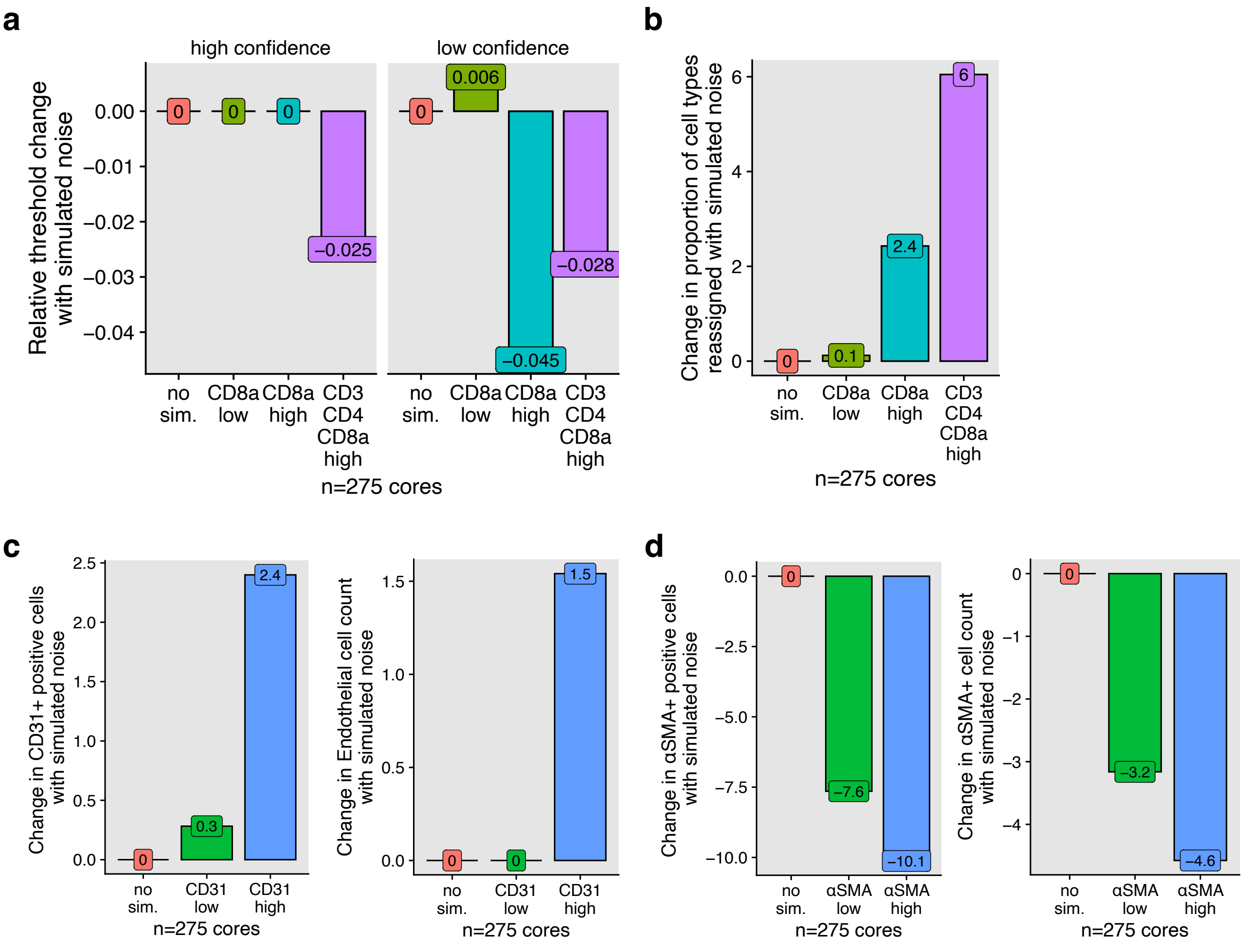


**Supplementary Figure 14**

**Supplementary Figure 14: Impact of different noise levels on the threshold estimate and change in cell population abundances.**

**a,** Changes in the low- and high-confidence group thresholds depending on the noise level (low and high) and markers with simulated noise (CD8a and CD3/CD4/CD8a). **b,** Proportion of cells that changed annotation due to the threshold change and simulated noise (3.16 million cells). **c-d,** Effect of different noise levels on cell positivity calls and cell type counts for two markers: CD31 **(c)** and αSMA **(d)**. Two levels of noise were simulated: low and high. The proportion of change in $CD31^{+}$/Endothelial cells and $αSMA^{+}$ cells was illustrated relative to the corresponding proportion based on raw intensities without noise simulation (no sim.), *i.e.* the percentage of cells that changed their positivity status with noise simulation over the total number without simulation. The analyses in **a-d** were performed on the T cells & Stroma panel.

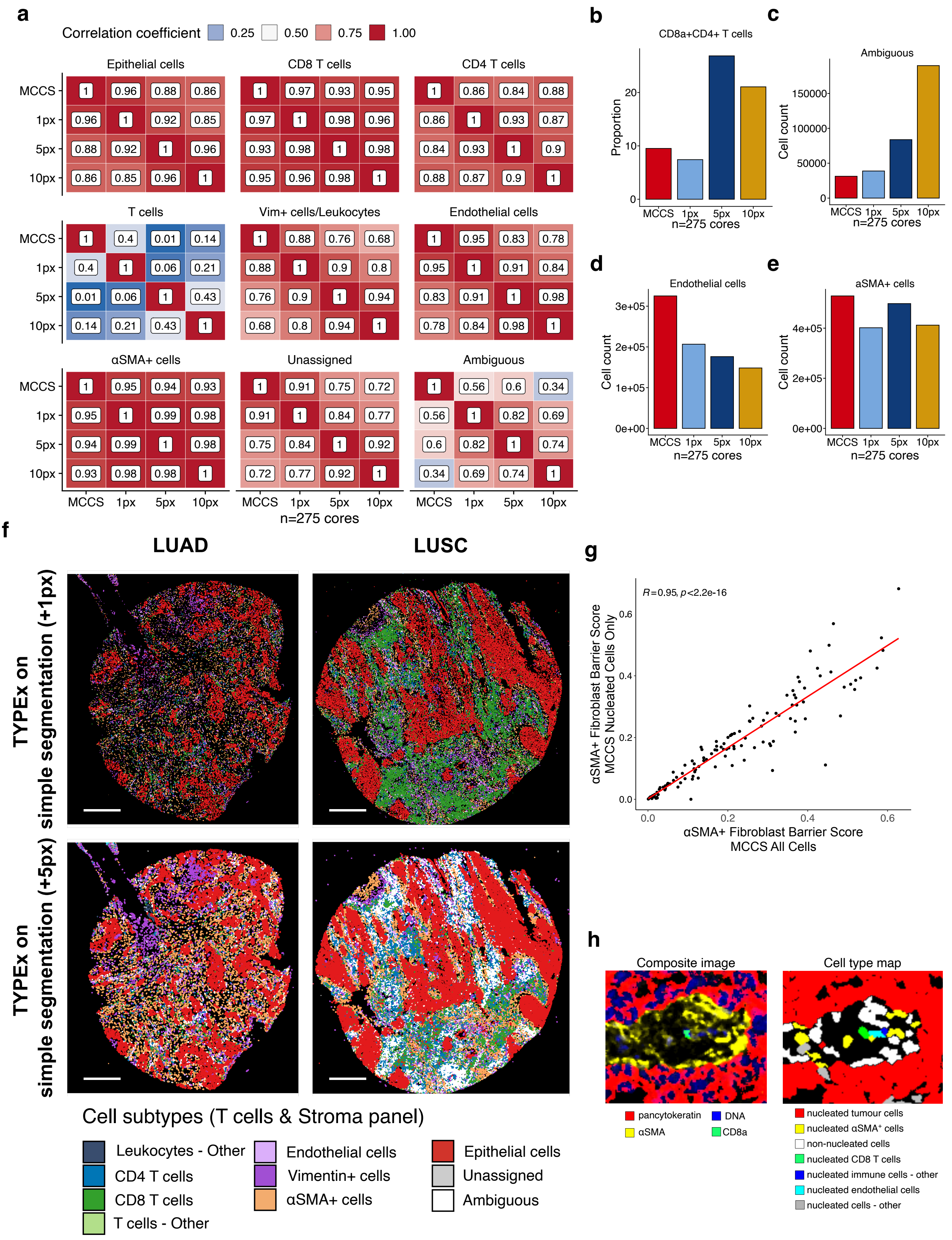


**Supplementary Figure 15**

**Supplementary Figure 15: Comparison of cell phenotyping and barrier scores between different segmentation approaches.**

Four segmentation approaches were compared: Multiplexed Consensus Cell Segmentation (MCCS) and cell segmentation with nuclear dilation of 1px, 5px and 10px using deep-imcyto in simple mode for the panel T cells & Stroma (n=275 cores, full dataset). Each of the segmentation outputs were then used as input to TYPEx for cell type analysis. **a,** Spearman correlation coefficients from pairwise comparison of the segmentation approaches for each major cell lineage. **b,** Comparison of the proportion of double-positive, CD8a$^{+}$CD4$^{+}$ T cells estimated with TYPEx using the four segmentation approaches as input. **c-e,** Comparison of the number of annotated cell types, Ambiguous **(c)**, Endothelial cells **(d)** and αSMA$^{+}$ cells **(e)** estimated with TYPEx based on the four segmentation approaches. **f,** Examples images with TYPEx cell classifications on cell objects derived from different segmentation approaches on the PHLEX test dataset. Two images from the test dataset show the classifications on cell objects segmented with simple segmentation with 1-pixel (px) (top) and 5-pixel (bottom) nuclear dilation. The results from the segmentation in MCCS mode for these images are shown in **Figure 4a**. Double-positive T cells are labelled Ambiguous. **g,** Spearman correlation for αSMA$^{+}$ fibroblast barrier scores (adjacent fraction mean score) for 121 LUAD and LUSC tumour cores in the TRACERx lung dataset, calculated using all cells identified using MCCS and only the nucleated cells identified using MCCS (R, Spearman correlation coefficient). **h,** Cropped area from a LUAD tumour core as preprocessed IMC composite (FIJI auto brightness/contrast adjustment + despeckling) (left) and MCCS cell type map (right). Source data are provided as a Source Data file. LUAD, lung adenocarcinoma; LUSC, lung squamous cell carcinoma; IMC, imaging mass cytometry.

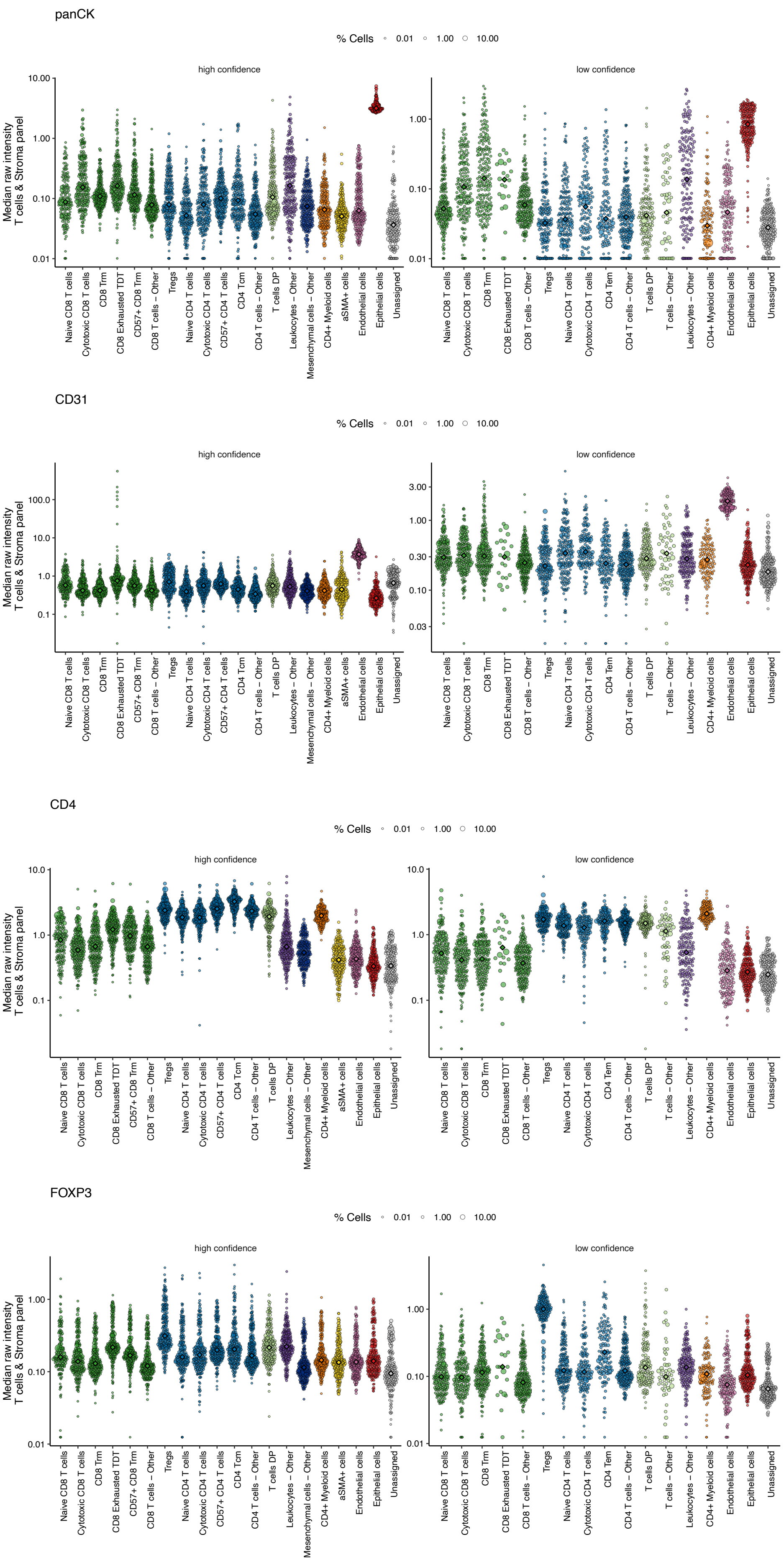


# Supplementary Figure 16

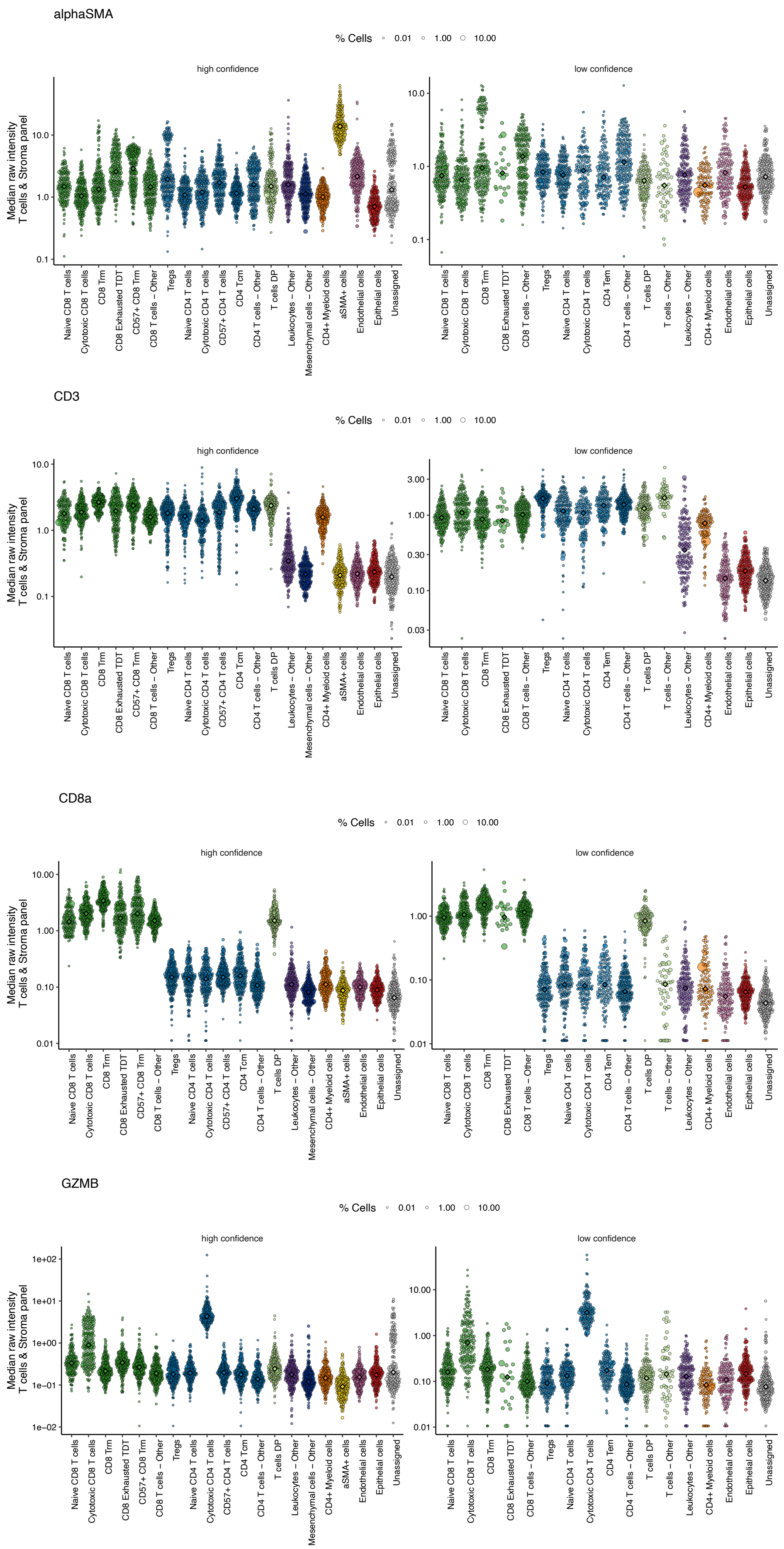


**Supplementary Figure 16 - continued**

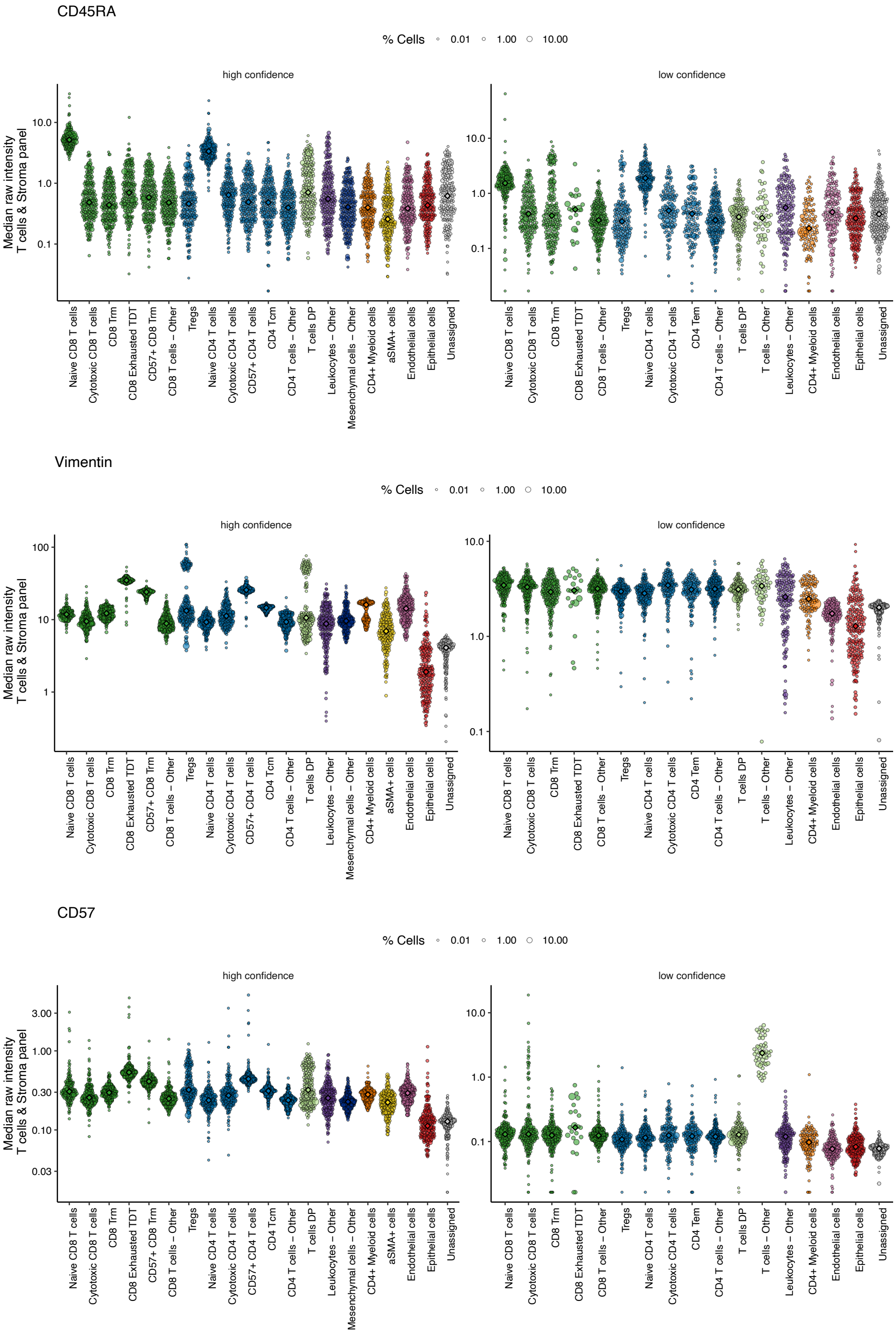


**Supplementary Figure 16 - continued**

**Supplementary Figure 16. Pixel intensity distributions for cell subtype-specific markers across different cell subtypes, split by the confidence group.**

Each point represents the median raw intensity of cells from a given cell subtype per image. Data from T cells & Stroma panel in the TRACERx 100 IMC cohort included. The diamond shape represents the median of all points. Source data are provided as a Source Data file. IMC, imaging mass cytometry.

## Supplementary Tables

**Supplementary Table 1. Imaging mass cytometry T cells & Stroma antibody panel used in the TRACERx 100 study.**

| T cells & Stroma IMC Panel | | | | | |
|---|---|---|---|---|---|
| **Protein** | **Metal** | **Company** | **Clone** | **Catalog No.** | **Dilution** |
| alphaSMA | 141Pr | Fluidigm | 1A4 | 3141017D | 1:250 |
| CCR7 | 142Nd | abcam | Y59 | ab221209 | 1:500 |
| Vimentin | 143Nd | Fluidigm | D21H3 | 3143027D | 1:100 |
| CD57 | 144Sm | abcam | HNK-1 or Leu-7 | ab212403 | 1:500 |
| CTLA4 | 145Nd | abcam | EPR1476 | ab209890 | 1:250 |
| FAP1 | 146Nd | sigma | SAB4500839 | SAB4500839 | 1:100 |
| CXCR6 | 147Sm | abcam | polyclonal | ab8023 | 1:100 |
| ICOS | 148Nd | Fluidigm | D1K2T | 3148021D | 1:100 |
| GATA3 | 149Sm | abcam | EPR16651 | ab214804 | 1:100 |
| PDL1 | 150Nd | abcam | SP142 | ab236238 | 1:100 |
| CD31 | 151Eu | Fluidigm | EPR3094 | 3151025D | 1:100 |
| CD45 | 152Sm | Fluidigm | 2B11 | 3152016D | 1:100 |
| LAG3 | 153Eu | abcam | EPR20261 | ab227579 | 1:100 |
| TIM3 | 154Sm | Fluidigm | D5D5R | 3154024D | 1:100 |
| FOXP3 | 155Gd | Fluidigm | 236A/ E7 | 3155016D | 1:100 |
| CD4 | 156Gd | Fluidigm | EPR6855 | 3156033D | 1:100 |
| CXCL12 | 158Gd | R&D | 79018 | MAB350-100 | 1:100 |
| CXCR4 | 159Tb | R&D | 12G5 | MAB170 | 1:100 |
| GITR | 160Gd | CST | D5V7P | 10419 | 1:100 |
| CD39 | 161Dy | abcam | EPR20627 | ab236038 | 1:100 |
| CD8a | 162Dy | Fluidigm | C8/144B | 3162034D | 1:500 |
| CD103 | 163Dy | abcam | EPR4166(2) | ab271889 | 1:500 |
| pancytokeratin | 164Dy | abcam | AE1/AE3 | ab80826 | 1:500 |
| PD1 | 165Ho | abcam | PDCD1/922 | ab215847 | 1:750 |
| CD45RA | 166Er | Fluidigm | HI100 | 3166028D | 1:1000 |
| GZMB | 167Er | Fluidigm | EPR20129-217 | 3167021D | 1:1000 |
| Ki67 | 168Er | Fluidigm | B56 | 3168022D | 1:250 |
| Collagen I | 169Tm | Fluidigm | polyclonal | 3169023D | 1:250 |
| CD3 | 170Er | Fluidigm | polyclonal | 3170019D | 1:100 |
| CD27 | 171Yb | Fluidigm | EPR8569 | 3171024D | 1:250 |
| cleaved-casp3 | 172Yb | Fluidigm | 5A1E | 3172027D | 1:100 |
| B2M | 173Yb | CST | D8P1H | 12851 | 1:100 |
| pSTAT1 | 174Yb | abcam | EPR3146 | ab215820 | 1:250 |
| CD25 | 175Lu | Fluidigm | EPR6452 | 3175036D | 1:200 |
| TCF1 | 176Yb | CST | C63D9 | 2203 | 1:500 |

**Supplementary Table 2. Imaging mass cytometry Pan-immune antibody panel used in the TRACERx 100 study.**

| Pan-immune IMC Panel | | | | | |
|---|---|---|---|---|---|
| **Antibody** | **Metal** | **Company** | **Clone** | **Catalog No.** | **Dilution** |
| CD38 | 141Pr | Fluidigm | EPR4106 | 3141018D | 1:100 |
| MPO | 142Nd | CST | E1E7I | 14569 | 1:250 |
| MCT4 | 143Nd | SantaCruz | D-1 | sc-376140 | 1:100 |
| CD14 | 144Sm | Fluidigm | EPR3653 | 3144025D | 1:100 |
| TCRd | 145Nd | SantaCruz | H-41 | sc-100289 | 1:100 |
| CD16 | 146Nd | Fluidigm | EPR16784 | 3146020D | 1:100 |
| CD163 | 147Sm | Fluidigm | EDHu-1 | 3147021D | 1:250 |
| KIR2DL3 | 148Nd | abcam | EPR22192 | ab241538 | 1:100 |
| CD11b | 149Sm | Fluidigm | EPR1344 | 3149028D | 1:500 |
| PDL1 | 150Nd | abcam | SP142 | ab236238 | 1:100 |
| CD31 | 151Eu | Fluidigm | EPR3094 | 3151025D | 1:100 |
| CD45 | 152Sm | Fluidigm | 2B11 | 3152016D | 1:100 |
| LAG3 | 153Eu | abcam | EPR20261 | ab227579 | 1:100 |
| TIM3 | 154Sm | Fluidigm | D5D5R | 3154024D | 1:100 |
| IDO | 155Gd | abcam | EPR20374 | ab224263 | 1:100 |
| CD4 | 156Gd | Fluidigm | EPR6855 | 3156033D | 1:100 |
| CD79a | 158Gd | abcam | EP3618 | ab239891 | 1:500 |
| CD68 | 159Tb | Fluidigm | KP1 | 3159035D | 1:250 |
| VISTA | 160Gd | Fluidigm | D1L2G | 3160025D | 1:500 |
| CD20 | 161Dy | Fluidigm | H1 | 3161029D | 1:500 |
| CD8a | 162Dy | Fluidigm | C8/144B | 3162034D | 1:500 |
| CD103 | 163Dy | abcam | EPR4166(2) | ab271889 | 1:500 |
| pancytokeratin | 164Dy | abcam | AE1/AE3 | ab80826 | 1:500 |
| PD1 | 165Ho | abcam | PDCD1/922 | ab215847 | 1:750 |
| CLEC9a | 166Er | Biolegend | LEC9A/DNGR | 353802 | 1:100 |
| GZMB | 167Er | Fluidigm | EPR20129-217 | 3167021D | 1:1000 |
| CD73 | 168Er | CST | D7F9A | 13160 | 1:250 |
| CD206 | 169Tm | CST | E2L9N | 91992 | 1:500 |
| CD3 | 170Er | Fluidigm | polyclonal | 3170019D | 1:100 |
| CD66b | 171Yb | abcam | polyclonal | ab218740 | 1:100 |
| CD56 | 172Yb | CST | 123C3 | 3576 | 1:50 |
| MHCII | 173Yb | abcam | 6C6 | ab55152 | 1:1000 |
| CD11c | 174Yb | LSBio | polyclonal | LS-A9381 | 1:250 |
| panactin | 175Lu | Fluidigm | D18C11 | 3175032D | 1:100 |
| CAIX | 176Yb | abcam | EPR4151(2) | ab180539 | 1:100 |

**Supplementary Table 3. Timing and computational requirements for cell phenotyping across different datasets.** Image width and height were determined as the range of X and Y cell coordinates, respectively, averaged across images.

| **dataset** | **TRACERx test dataset** | **HuBMAP** | **BE** | **TRACERx 100 (T cells & Stroma)** | **Schürch *et al.*** |
|---|---|---|---|---|---|
| **# cells** | 139,911 | 248,285 | 219,926 | 3,158,829 | 258,385 |
| **# markers** | 35 | 48 | 44 | 35 | 58 |
| **# cell lineage markers** | 8 | 24 | 21 | 8 | 16 |
| **number of images** | 5 | 8 | 2 | 277 | 140 |
| **image width** | 2333 | 9280 | 9404 | 1831 | 1830 |
| **image height** | 2179 | 8989 | 9067 | 1716 | 1420 |
| **segmentation input** | deep-imcyto (MCCS mode) | user-provided cell objects table | user-provided cell objects table | deep-imcyto (MCCS mode) | user-provided cell objects table |
| **multiplexed imaging** | IMC | CODEX | CODEX | IMC | CODEX |
| **tissue type** | NSCLC and lymph node | Barrett's esophagus and tonsil | healthy intestine | NSCLC, tonsil, lymph node | colorectal cancer |
| **tissue processing** | TMA | fresh frozen | fresh frozen | TMA | TMA |
| **max CPUs** | 4 | 27 | 27 | 25 | 27 |
| **max memory (GB)** | 6 | 44 | 31 | 98 | 37 |
| **duration[a]** | 3h 22m[b] (2h 40m[c]) | 5h 52m | 7h 24m | 14h 30m (9h 45m[c]) | 15h 45m[c] |

[a] Queueing time not considered

[b] Similar timing for deep-imcyto input in *CellProfiler* mode (MCCS procedure) and *simple* segmentation (1px and 5px dilation).

[c] Complete and incomplete models run in parallel (--*excluded_cell_lineage* argument specified)

**Supplementary Table 4. Segmentation markers chosen for TRACERx 100 antibody panels for the MCCS segmentation procedure implemented in deep-imcyto *CellProfiler* mode.** Marker indicates the protein target, panel(s) refers to whether that marker was used for segmentation in one or both of the Pan-Immune or T cells & Stroma antibody panels and the cell types expected to express the marker are listed.

| Marker | Panel(s) | Cell types expressing the marker |
|---|---|---|
| CD8a | T cells & Stroma, Pan-Immune | CD8a+ T cells |
| CD4 | T cells & Stroma, Pan-Immune | CD4+ T cells |
| TCRd | Pan-Immune | Gamma-delta T cells |
| CD3 | T cells & Stroma, Pan-Immune | T cells |
| CD11b | Pan-Immune | Myeloid cells |
| CD66b | Pan-Immune | Granulocytes, including neutrophils |
| CD20 | Pan-Immune | B cells |
| CD79a | Pan-Immune | B cells and plasma cells |
| CD206 | Pan-Immune | Macrophages |
| CD163 | Pan-Immune | Macrophages |
| CD68 | Pan-Immune | Macrophages |
| CD16 | Pan-Immune | Myeloid cells and NK cells |
| CD45 | T cells & Stroma, Pan-Immune | Leukocytes |
| pancytokeratin | T cells & Stroma, Pan-Immune | Tumour and normal epithelial cells |
| CD31 | T cells & Stroma, Pan-Immune | Endothelial cells |
| CD14 | Pan-Immune | Monocytes and myeloid cells |
| aSMA | T cells & Stroma | Fibroblasts, smooth muscle, pericytes |
| vimentin | T cells & Stroma | Mesenchymal tumour, stromal and immune cells |
| panactin | Pan-Immune | All cells |

**Supplementary Table 5. List of protein markers used for tissue segmentation.** Immune and stromal markers were combined to represent the Stroma tissue compartment, and pancytokeratin was used for the Tumour/Epithelial tissue compartment. A composite image was created using a distinct pseudo-color for the combined immune/stromal markers, pancytokeratin and combined DNA channels.

| Marker protein | Tissue compartment | Antibody panel | Marker protein expression | Metal |
|---|---|---|---|---|
| panCK | Tumour | T cells & Stroma,Pan Immune | tumour | 164Dy |
| CD45 | Stroma | T cells & Stroma,Pan Immune | stroma - immune | 152Sm |
| CD4 | Stroma | T cells & Stroma,Pan Immune | stroma - immune | 156Gd |
| CD45 | Stroma | T cells & Stroma,Pan Immune | stroma - immune | 152Sm |
| CD206 | Stroma | Pan Immune | stroma - immune | 169Tm |
| CD20 | Stroma | Pan Immune | stroma - immune | 161Dy |
| CD11b | Stroma | Pan Immune | stroma - immune | 149Sm |
| CD79a | Stroma | Pan Immune | stroma - immune | 158Gd |
| CD163 | Stroma | Pan Immune | stroma - immune | 147Sm |
| CD16 | Stroma | Pan Immune | stroma - immune | 146Nd |
| CD68 | Stroma | Pan Immune | stroma - immune | 159Tb |
| CD14 | Stroma | Pan Immune | stroma - immune | 144Sm |
| CD4 | Stroma | T cells & Stroma,Pan Immune | stroma - immune | 156Gd |
| CD3 | Stroma | T cells & Stroma,Pan Immune | stroma - immune | 170Er |
| CD8a | Stroma | T cells & Stroma,Pan Immune | stroma - immune | 162Dy |
| aSMA | Stroma | T cells & Stroma | stroma - non-immune | 141Pr |
| CD31 | Stroma | T cells & Stroma,Pan Immune | stroma - non-immune | 151Eu |
| vimentin | Stroma | T cells & Stroma | stroma (tumour excluded) | 143Nd |
| collagen1 | Stroma | T cells & Stroma | stroma (tumour excluded) | 169Tm |
| panactin | Stroma | Pan Immune | stroma (tumour excluded) | 175Lu |
| DNA1 | / | T cells & Stroma,Pan Immune | DNA | 191Ir |
| DNA2 | / | T cells & Stroma,Pan Immune | DNA | 193Ir |

# References


1. Catena, R., Montuenga, L. M. & Bodenmiller, B. Ruthenium counterstaining for imaging mass cytometry. *J. Pathol.* **244**, 479–484 (2018).
2. Zhou, Z., Siddiquee, M. M. R., Tajbakhsh, N. & Liang, J. UNet++: Redesigning Skip Connections to Exploit Multiscale Features in Image Segmentation. *IEEE Trans. Med. Imaging* **39**, 1856–1867 (2020).
3. Zhang, A. W. *et al.* Probabilistic cell-type assignment of single-cell RNA-seq for tumor microenvironment profiling. *Nat. Methods* **16**, 1007–1015 (2019).
4. Van Gassen, S. *et al.* FlowSOM: Using self-organizing maps for visualization and interpretation of cytometry data. *Cytometry Part A* vol. 87 636–645 Preprint at https://doi.org/10.1002/cyto.a.22625 (2015).
5. Bodenheimer, T. *et al.* FastPG: Fast clustering of millions of single cells. *bioRxiv* 2020.06.19.159749 (2020) doi:10.1101/2020.06.19.159749.
6. Levine, J. H. *et al.* Data-Driven Phenotypic Dissection of AML Reveals Progenitor-like Cells that Correlate with Prognosis. *Cell* **162**, 184–197 (2015).
7. Lu, P. *et al.* IMC-Denoise: a content aware denoising pipeline to enhance Imaging Mass Cytometry. *Nat. Commun.* **14**, 1601 (2023).
8. Schürch, C. M. *et al.* Coordinated Cellular Neighborhoods Orchestrate Antitumoral Immunity at the Colorectal Cancer Invasive Front. *Cell* **183**, 838 (2020).
9. Brbić, M. *et al.* Annotation of spatially resolved single-cell data with STELLAR. *Nat. Methods* **19**, 1411–1418 (2022).
10. Greenwald, N. F. *et al.* Whole-cell segmentation of tissue images with human-level performance using large-scale data annotation and deep learning. *Nat. Biotechnol.* **40**, 555–565 (2022).
11. van Maldegem, F. *et al.* Characterisation of tumour microenvironment remodelling following oncogene inhibition in preclinical studies with imaging mass cytometry. *Nat. Commun.* **12**, 5906 (2021).

12. Jackson, H. W. *et al.* The single-cell pathology landscape of breast cancer. *Nature* vol. 578 615–620 Preprint at https://doi.org/10.1038/s41586-019-1876-x (2020).
