# Supplementary material for: Deep cell phenotyping and spatial analysis of multiplexed imaging with TRACERx-PHLEX
Source: Nat Commun. 2024 Jun 15;15:5135. doi: 10.1038/s41467-024-48870-5 (PMC11180132; doi:10.1038/s41467-024-48870-5)
Supplement: Supplementary file 2 — Reporting Summary [file 41467_2024_48870_MOESM2_ESM.pdf]

nature portfolio

Corresponding author(s): Charles Swanton

Last updated by author(s): May 2, 2024

Reporting Summary

Nature Portfolio wishes to improve the reproducibility of the work that we publish. This form provides structure for consistency and transparency in reporting. For further information on Nature Portfolio policies, see our Editorial Policies and the Editorial Policy Checklist.

Statistics

For all statistical analyses, confirm that the following items are present in the figure legend, table legend, main text, or Methods section.

n/a	Confirmed	
[ ]	[x]	The exact sample size (*n*) for each experimental group/condition, given as a discrete number and unit of measurement
[ ]	[x]	A statement on whether measurements were taken from distinct samples or whether the same sample was measured repeatedly
[ ]	[x]	The statistical test(s) used AND whether they are one- or two-sided<br>*Only common tests should be described solely by name; describe more complex techniques in the Methods section.*
[ ]	[x]	A description of all covariates tested
[ ]	[x]	A description of any assumptions or corrections, such as tests of normality and adjustment for multiple comparisons
[ ]	[x]	A full description of the statistical parameters including central tendency (e.g. means) or other basic estimates (e.g. regression coefficient) AND variation (e.g. standard deviation) or associated estimates of uncertainty (e.g. confidence intervals)
[ ]	[x]	For null hypothesis testing, the test statistic (e.g. *F*, *t*, *r*) with confidence intervals, effect sizes, degrees of freedom and *P* value noted<br>*Give P values as exact values whenever suitable.*
[x]	[ ]	For Bayesian analysis, information on the choice of priors and Markov chain Monte Carlo settings
[x]	[ ]	For hierarchical and complex designs, identification of the appropriate level for tests and full reporting of outcomes
[ ]	[x]	Estimates of effect sizes (e.g. Cohen's *d*, Pearson's *r*), indicating how they were calculated

Our web collection on statistics for biologists contains articles on many of the points above.

Software and code

Policy information about availability of computer code

Data collection	Imaging mass cytometry data were acquired using a Hyperion Imaging System using commercial Standard Biotools/Fluidigm imaging mass cytometry software (version 6.7).
Data analysis	TRACERx-PHLEX pipeline runs using publicly available docker containers, which incorporate all specific software packages required to reproduce the reported results:<br><br>magnesa/phlex:deepimcyto_4<br>quay.io/biocontainers-imctools-1.0.5--py_0<br>quay.io/biocontainers/r-rmarkdown:0.9.5--r3.3.2_0<br>cellprofiler/cellprofiler:3.1.9<br>mihangelova/typer:2.7<br>mihangelova/typefiji:latest<br>mihangelova/typeconda:latest<br>mihangelova/visr:latest<br>ilastik/ilastik-from-binary:1.4.0b13<br><br>Code repository:<br>https://github.com/FrancisCrickInstitute/TRACERx-PHLEX<br><br>The deep-imcyto core nuclear prediction model is available for users who wish to use it outside of the deep-imcyto Nextflow pipeline:

https://github.com/FrancisCrickInstitute/py-imcyto

These pipelines are executed with
Nextflow (version 22.04.0)
Singularity (version 3.6.4)

Data visualisation, image and neighbourhood analysis
R (version 3.6.3 or later 4.1.1 and 4.2.0)
Python (version 2.7.12, 3.8.3 and 3.10.1)
CellProfiler software (v3.1.9)
QuPath v0.3.2
ggplot2 (version 3.3.2, 3.3.6)

Statistics
nlme version(3.1.153, 3.1.159)
lme4 (version 1.1.30)
ROCR (version 1.0-11)

For manuscripts utilizing custom algorithms or software that are central to the research but not yet described in published literature, software must be made available to editors and reviewers. We strongly encourage code deposition in a community repository (e.g. GitHub). See the Nature Portfolio guidelines for submitting code & software for further information.

Data

Policy information about availability of data

All manuscripts must include a data availability statement. This statement should provide the following information, where applicable:

- Accession codes, unique identifiers, or web links for publicly available datasets
- A description of any restrictions on data availability
- For clinical datasets or third party data, please ensure that the statement adheres to our policy

The TRACERx Nuclear IMC segmentation dataset and deep-imcyto's trained neural network model weights can be downloaded from Zenodo under accession code 7973724. A testing dataset consisting of five samples from the TRACERx IMC data, panel T cells & Stroma has been deposited on Zenodo under accession code 7973724 and the TRACERx-PHLEX GitHub repository.

The raw imaging mass cytometry data generated in Enfield et al. and analysed in this study are available under restricted access due to privacy through the CRUK and UCL Cancer Trials Centre (ctc.tracerx@ucl.ac.uk) for academic non-commercial research purposes. Access will be granted upon review of a project proposal, which will be evaluated by a TRACERx data access committee and entered into an appropriate data access agreement, subject to any applicable ethical approvals. WES data from the TRACERx cohort included in this study have been deposited at the European Genome–phenome Archive (EGA) with details on how to apply for access. The data are hosted by The European Bioinformatics Institute and the Centre for Genomic Regulation (CRG) under the accession code EGAS00001006494 (WES); access is controlled by the TRACERx data access committee.

For the publicly available colorectal cancer CODEX dataset, the cell-by-marker intensity table and the published cell-type annotations were available under DOI: 10.17632/mpjzbtfgfr.1, and the manual gating information was obtained from CellEngine under accession number 5ea1170788ae4203c2959042. The CODEX datasets on healthy intestine (HuBMAP) and Barrett's oesophagus (BE) together with the associated ground truth annotations were provided on the online repository Dryad https://datadryad.org/stash/share/1OQtxew0Unh3iAdP-ELew-ctwuPTBz6Oy8uuyxqliZk.

Raw tiff files used in this study for validation and benchmarking of the segmentation approach are available on a Figshare repository under the handle 10779/crick.c.5270621.v2 for the mouse IMC data from lung cancer, on Zenodo under accession code 3518284 for the human breast cancer IMC data, and on datasets.deepcell.org for TissueNet breast cancer data.

The data generated and visualised in this study are provided as Source Data files.

Research involving human participants, their data, or biological material

Policy information about studies with human participants or human data. See also policy information about sex, gender (identity/presentation), and sexual orientation and race, ethnicity and racism.

Reporting on sex and gender	Sex annotations are available for all patients in this TRACERx study and their distributions outlined below. Sex was not an inclusion or exclusion criteria in this study. Our imaging mass cytometry cohort is comprised of one third females and two thirds males.
Reporting on race, ethnicity, or other socially relevant groupings	Ethnicity annotations are available for all patients in this TRACERx IMC study, and was not an inclusion or exclusion criteria. 96% of the cohort is from a white ethnic background.
Population characteristics	Considering both IMC antibody panels, our PHLEX cohort of 83 TRACERx patients was comprised of 159 tumour regions from 73 patients (p1: 164 images, 144 regions, 72 patients; p2: 169 images, 150 regions, 71 patients), 21 benign tumour-adjacent regions from 17 patients (p1: 24 images, 21 regions, 17 patients; p2: 19 images, 17 regions, 13 patients), five lymph node regions with no tumour content (p1: 5 regions, 5 patients; p2, 4 regions, 4 patients), one lymph node region with tumour content (p1 and p2), 49 adjacent normal lung cores from 49 patients (p1: 50 images, 46 regions, 46 patients; p2: 51 images, 46 regions, 46 patients), tonsil controls (p1: 29 images; p2: 33 images), and one kidney control (p1 and p2). Further, we

included data from a single tumour region (p1 and p2) however we were unable to map to patient metadata. Multiple images per region resulted from technical replicates or where multiple TMA cores were taken from the same regional FFPE block and inserted into independent TMAs.

33.3% are females, 66.7% are males; 91% are smokers or have a smoking history, 9% are never smokers; 21% of patients were diagnosed at stage IA, 35% at IB, 16% at IIA, 12% at IIB, 15% at IIIA, and 1% at IIIB; 57% of diagnosed tumours were adenocarcinomas, 36% were squamous cell carcinomas and 7% were of other histological subtypes; 96% of the cohort is from a white ethnic background and the mean age of the patients is 68, ranging between 34 and 85.

Please note that the study started recruiting patients in 2016, when TNM version 7 was standard of care. The up-to-date inclusion/exclusion criteria now utilizes TNM version 8.

TRACERx inclusion and exclusion criteria

Inclusion Criteria:
_Written Informed consent
_Patients ≥18 years of age, with early stage I-IIIB disease (according to TNM 8th edition) who are eligible for primary surgery.
_Histopathologically confirmed NSCLC, or a strong suspicion of cancer on lung imaging necessitating surgery (e.g. diagnosis determined from frozen section in theatre)
_Primary surgery in keeping with NICE guidelines planned
_Agreement to be followed up at a TRACERx site
_Performance status 0 or 1
_Minimum tumor diameter at least 15mm to allow for sampling of at least two tumour regions (if 15mm, a high likelihood of nodal involvement on pre-operative imaging required to meet eligibility according to stage, i.e. T1N1-3)

Exclusion Criteria:
_Any other* malignancy diagnosed or relapsed at any time, which is currently being treated (including by hormonal therapy).
_Any other* current malignancy or malignancy diagnosed or relapsed within the past 3 years**.
*Exceptions are: non-melanomatous skin cancer, stage 0 melanoma in situ, and in situ cervical cancer
**An exception will be made for malignancies diagnosed or relapsed more than 2, but less than 3, years ago only if a pre-operative biopsy of the lung lesion has confirmed a diagnosis of NSCLC.
_Psychological condition that would preclude informed consent
_Treatment with neo-adjuvant therapy for current lung malignancy deemed necessary
_Post-surgery stage IV
_Known Human Immunodeficiency Virus (HIV), Hepatitis B Virus (HBV), Hepatitis C Virus (HCV) or syphilis infection.
_Sufficient tissue, i.e. a minimum of two tumor regions, is unlikely to be obtained for the study based on pre-operative imaging

Patient ineligibility following registration
_There is insufficient tissue
_The patient is unable to comply with protocol requirements
_There is a change in histology from NSCLC following surgery, or NSCLC is not confirmed during or after surgery.
_Change in staging to IIIC or IV following surgery
_The operative criteria are not met (e.g. incomplete resection with macroscopic residual tumors (R2)). Patients with microscopic residual tumors (R1) are eligible and should remain in the study
_Adjuvant therapy other than platinum-based chemotherapy and/or radiotherapy is administered.

Recruitment

When patients are initially diagnosed with stage I-III lung cancer and then referred for surgical resection, a research nurse identifies them on a clinic/operating list. The patient has an initial eligibility assessment and is then provided with written information about the TRACERx study and he/she can ask the research nurse any questions.

Patients have to agree to provide serial blood samples whenever they attend clinic for routine blood sampling, so this represents the only main potential self-selecting bias (i.e. only patients willing to do this would participate). However, it is unclear how this would affect the biomarker analyses. Also, the gender and ethnicity characteristics are in line with patients seen in routine practice.

Inclusion and exclusion criteria are summarised above.

All patients were assigned a study ID that was known to the patient. These were subsequently converted to linked study IDs such that the patients could not identify themselves in study publications. All human samples, tissue and blood, were linked to the study ID and barcoded such that they were anonymised and tracked on a centralised database overseen by the study sponsor only. Written informed consent was obtained from all patients.

Ethics oversight

The TRACERx study was approved by the National Research Ethics Service Committee London, with sponsor's approval of the study by University College London (UCL), with the following details:
Study title: TRAcking non small cell lung Cancer Evolution through therapy (Rx)
REC reference: 13/LO/1546
Protocol number: UCL/12/0279
IRAS project ID: 138871

Use of an additional human FFPE sample from the UCL/UCLH Biobank for Studying Health and Disease was covered under

ethics approval 20/YH/0088, UCL/UCLH Biobank for Studying Health and Disease Renewal 2020.

Note that full information on the approval of the study protocol must also be provided in the manuscript.

Field-specific reporting

Please select the one below that is the best fit for your research. If you are not sure, read the appropriate sections before making your selection.

- [x] Life sciences
- [ ] Behavioural & social sciences
- [ ] Ecological, evolutionary & environmental sciences

For a reference copy of the document with all sections, see nature.com/documents/nr-reporting-summary-flat.pdf

Life sciences study design

All studies must disclose on these points even when the disclosure is negative.

Sample size

The imaging mass cytometry cohort involves a subset of the TRACERx 100 cohort. The sample size of 83 patients represents those patients for which sufficient FFPE material for immunostaining was available and that was of sufficient quality for analysis.

TRACERx is a programme of work of multiple projects built around a single observational cohort study. It is not possible to perform a sample size calculation for each project, especially post hoc. The size of the full TRACERx longitudinal study was done in relation to tumour heterogeneity and disease free survival.

Data exclusions

Imaging mass cytometry data which fail quality control derived from pathology review were excluded from analysis.

Replication

TRACERx is a prospective longitudinal study. As such, the results shown here are not the result of an experimental set up. Imaging mass cytometry data corresponds to the first 100 patients, while validation data was derived from the half-way point of the TRACERx study (421 patients total) and reflects hypothesis generating analysis.

Randomization

Randomization is not relevant as this is an observational study.

Blinding

Blinding is not relevant as this is an observational study. Patients were not allocated to any intervention and they were followed up and assessed as per routine practice. No biomarker results (tissue and bloods) are reported back to patients, so there is no likelihood of people changing their behaviours based on these findings. The laboratory analyses were all performed without knowing the outcome (DFS or survival) status of the patients, which represents a form of blinding.

Reporting for specific materials, systems and methods

We require information from authors about some types of materials, experimental systems and methods used in many studies. Here, indicate whether each material, system or method listed is relevant to your study. If you are not sure if a list item applies to your research, read the appropriate section before selecting a response.

Materials & experimental systems

n/a	Involved in the study	
[ ]	[x]	Antibodies
[x]	[ ]	Eukaryotic cell lines
[x]	[ ]	Palaeontology and archaeology
[x]	[ ]	Animals and other organisms
[ ]	[x]	Clinical data
[x]	[ ]	Dual use research of concern
[x]	[ ]	Plants

Methods

n/a	Involved in the study	
[x]	[ ]	ChIP-seq
[x]	[ ]	Flow cytometry
[x]	[ ]	MRI-based neuroimaging

Antibodies

Antibodies used

Information of the antibodies used in this study can be found in Supplementary Table 1 and are listed below. Custom-conjugations involving different antibody lots were titrated before use.

alphaSMA(1A4)-141Pr Fluidigm Cat No. 3141017D dilution 1:250
CCR7(Y59)-142Nd abcam Cat No. ab221209 dilution 1:500
Vimentin(D21H3)-143Nd Fluidigm Cat No. 3143027D dilution 1:100
CD57(HNK-1 or Leu-7)-144Sm abcam Cat No. ab212403 dilution 1:500
CTLA4(EPR1476)-145Nd abcam Cat No. ab209890 dilution 1:250
FAP1(SAB4500839)-146Nd sigma Cat No. SAB4500839 dilution 1:100
CXCR6(polyclonal)-147Sm abcam Cat No. ab8023 dilution 1:100
ICOS(D1K2T)-148Nd Fluidigm Cat No. 3148021D dilution 1:100
GATA3(EPR16651)-149Sm abcam Cat No. ab214804 dilution 1:100

PDL1(SP142)-150Nd abcam Cat No. ab236238 dilution 1:100
CD31(EPR3094)-151Eu Fluidigm Cat No. 3151025D dilution 1:100
CD45(2B11)-152Sm Fluidigm Cat No. 3152016D dilution 1:100
LAG3(EPR20261)-153Eu abcam Cat No. ab227579 dilution 1:100
TIM3(D5D5R)-154Sm Fluidigm Cat No. 3154024D dilution 1:100
FOXP3(236A/ E7)-155Gd Fluidigm Cat No. 3155016D dilution 1:100
CD4(EPR6855)-156Gd Fluidigm Cat No. 3156033D dilution 1:100
CXCL12(79018)-158Gd R&D Cat No. MAB350-100 dilution 1:100
CXCR4(12G5)-159Tb R&D Cat No. MAB170 dilution 1:100
GITR(D5V7P)-160Gd CST Cat No. 10419 dilution 1:100
CD39(EPR20627)-161Dy abcam Cat No. ab236038 dilution 1:100
CD8a(C8/144B)-162Dy Fluidigm Cat No. 3162034D dilution 1:500
CD103(EPR4166(2))-163Dy abcam Cat No. ab271889 dilution 1:500
pancytokeratin(AE1/AE3)-164Dy abcam Cat No. ab80826 dilution 1:500
PD1(PDCD1/922)-165Ho abcam Cat No. ab215847 dilution 1:750
CD45RA(HI100)-166Er Fluidigm Cat No. 3166028D dilution 1:1000
GZMB(EPR20129-217)-167Er Fluidigm Cat No. 3167021D dilution 1:1000
Ki67(B56)-168Er Fluidigm Cat No. 3168022D dilution 1:250
Collagen I(polyclonal)-169Tm Fluidigm Cat No. 3169023D dilution 1:250
CD3(polyclonal)-170Er Fluidigm Cat No. 3170019D dilution 1:100
CD27(EPR8569)-171Yb Fluidigm Cat No. 3171024D dilution 1:250
cleaved-casp3(5A1E)-172Yb Fluidigm Cat No. 3172027D dilution 1:100
B2M(D8P1H)-173Yb CST Cat No. 12851 dilution 1:100
pSTAT1(EPR3146)-174Yb abcam Cat No. ab215820 dilution 1:250
CD25(EPR6452)-175Lu Fluidigm Cat No. 3175036D dilution 1:200
TCF1(C63D9)-176Yb CST Cat No. 2203 dilution 1:500
CD38(EPR4106)-141Pr Fluidigm Cat No. 3141018D dilution 1:100
MPO(E1E7I)-142Nd CST Cat No. 14569 dilution 1:250
MCT4(D-1)-143Nd SantaCruz Cat No. sc-376140 dilution 1:100
CD14(EPR3653)-144Sm Fluidigm Cat No. 3144025D dilution 1:100
TCRd(H-41)-145Nd SantaCruz Cat No. sc-100289 dilution 1:100
CD16(EPR16784)-146Nd Fluidigm Cat No. 3146020D dilution 1:100
CD163(EDHu-1)-147Sm Fluidigm Cat No. 3147021D dilution 1:250
KIR2DL3(EPR22192)-148Nd abcam Cat No. ab241538 dilution 1:100
CD11b(EPR1344)-149Sm Fluidigm Cat No. 3149028D dilution 1:500
IDO(EPR20374)-155Gd abcam Cat No. ab224263 dilution 1:100
CD79a(EP3618)-158Gd abcam Cat No. ab239891 dilution 1:500
CD68(KP1)-159Tb Fluidigm Cat No. 3159035D dilution 1:250
VISTA(D1L2G)-160Gd Fluidigm Cat No. 3160025D dilution 1:500
CD20(H1)-161Dy Fluidigm Cat No. 3161029D dilution 1:500
CLEC9a(CLEC9A/DNGR1)-166Er Biolegend Cat No. 353802 dilution 1:100
CD73(D7F9A)-168Er CST Cat No. 13160 dilution 1:250
CD206(E2L9N)-169Tm CST Cat No. 91992 dilution 1:500
CD66b(polyclonal)-171Yb abcam Cat No. ab218740 dilution 1:100
CD56(123C3)-172Yb CST Cat No. 3576 dilution 1:50
MHCII(6C6)-173Yb abcam Cat No. ab55152 dilution 1:1000
CD11c(polyclonal)-174Yb LSBio Cat No. LS-A9381 dilution 1:250
panactin(D18C11)-175Lu Fluidigm Cat No. 3175032D dilution 1:100
CAIX(EPR4151(2))-176Yb abcam Cat No. ab180539 dilution 1:100

Validation

All antibodies were selected for application on human FFPE tissues and tested in-house prior to inclusion in the final antibody panels. Antibodies were validated by multicolour immunofluorescence and imaging mass cytometry using markers of expected co-expression or mutual exclusivity in tonsil, lung cancer and normal lung human FFPE tissues as well as positive and negative controls (lung cancer, normal tonsil, normal cardiac, normal brain tissue). The complete, metal-conjugated antibody panels were further validated using the same control tissues and a dilution series to test for quality and specificity of staining.

Antibody manufacturer descriptions of technical validation can be found on suppliers' websites. Antibodies available from Standard Biotools/Fluidigm state the following on their Technical Data Sheets: “MaxPar® OnDemand Antibodies are a curated collection of antibody clones that have been tested by Imaging Mass Cytometry in biologically relevant tissues. A variety of metal options have been selected to optimise panel design for flexibility and performance.”

Clinical data

Policy information about clinical studies

All manuscripts should comply with the ICMJE guidelines for publication of clinical research and a completed CONSORT checklist must be included with all submissions.

Clinical trial registration	TRACERx Lung https://clinicaltrials.gov/ct2/show/NCT01888601, approved by an independent Research Ethics Committee, 13/LO/1546
Study protocol	https://clinicaltrials.gov/ct2/show/NCT01888601
Data collection	Clinical and pathological data is collected from patients during study follow up - this period is a minimum of five years. Data collection is overseen by the sponsor of the study (Cancer Research UK & UCL Cancer Trials Centre) and takes place in hospitals across the United Kingdom. A centralised database called MACRO is used for this purpose. Recruitment started in April 2014 and is still ongoing (in London and Manchester).
Outcomes	The main clinical outcomes are:<br>Disease-free survival (DFS) – measured from the time of study registration to date of first lung recurrence or death from any cause. Patients who do not have these events are censored at the date last known to be alive (including patients who developed a new primary tumour that has been shown biologically to not be linked to the initial primary lung tumour).<br><br>In this paper, lung cancer specific survival metrics were also used to assess risk of lung cancer-related death or relapse.<br><br>TRACERx primary outcome: determine the clinical impact of intratumour heterogeneity on the clinical course of disease and the impact of adjuvant platinum-based chemo on intratumour heterogeneity in relapsed disease.<br><br>TRACERx secondary outcome: No secondary outcome was pre-defined.

Plants

Seed stocks	N/A
Novel plant genotypes	N/A
Authentication	N/A
